# Immigrant women’s experiences of maternity-care services in Canada: a systematic review using a narrative synthesis

**DOI:** 10.1186/2046-4053-4-13

**Published:** 2015-02-11

**Authors:** Gina MA Higginbottom, Myfanwy Morgan, Mirande Alexandre, Yvonne Chiu, Joan Forgeron, Deb Kocay, Rubina Barolia

**Affiliations:** Faculty of Nursing, Edmonton Clinic Health Academy, University of Alberta, Edmonton, AB T6G 1C9 Canada; King’s College London, Primary Care and Public Health Sciences London, London, SE1 3QD UK; Citizenship and Immigration Canada, New Multiculturalism Grants and Contributions Program, Canada Place, Edmonton, AB T5J 4C3 Canada; Multicultural Health Brokers Co-operative, Edmonton, AB T5H 2M6 Canada; Lois Hole Hospital, Alberta Health Services, Edmonton, AB T5H 3V9 Canada; Health Canada, Public Health Agency of Canada, Calgary, AB T2G 4X3 Canada

**Keywords:** Immigrant women, Maternity-care experience, Canada, Systematic review, Narrative synthesis, Postpartum depression

## Abstract

**Background:**

Canada’s diverse society and its statutory commitment to multiculturalism means that a synthesis of knowledge related to the healthcare experiences of immigrants is essential to realise the health potential for future Canadians. Although concerns about the maternity experiences of immigrants in Canada are relatively new, recent national guidelines explicitly call for the tailoring of services to user needs. We therefore assessed the experiences of immigrant women accessing maternity-care services in Canada. In particular, we investigated the experiences of immigrant women in Canada in accessing and navigating maternity and related healthcare services from conception to 6 months postpartum in Canada. Our focus was on (*a*) the accessibility and acceptability of maternity-care services for immigrant women and (*b*) the effects of the perceptions and experiences of these women on their birth and postnatal outcomes.

**Methods:**

We conducted a systematic review using a systematic search and narrative synthesis of peer-reviewed and non-peer-reviewed reports of empirical research, with the aim of providing stakeholders with perspectives on maternity-care services as experienced by immigrant women. We partnered with key stakeholders (‘integrated knowledge users’) to ensure the relevancy of topics and to tailor recommendations for effective translation into future policy, practice and programming. Two search phases and a three-stage selection process for published and grey literature were conducted prior to appraisal of literature quality and narrative synthesis of the findings.

**Results:**

Our knowledge synthesis of maternity care among immigrants to Canada provided a coherent evidence base for (*a*) eliciting a better understanding of the factors that generate disparities in accessibility, acceptability and outcomes during maternity care; and (*b*) improving culturally based competency in maternity care. Our synthesis also identified pertinent issues in multiple sectors that should be addressed to configure maternity services and programs appropriately.

**Conclusions:**

Although immigrant women in Canada are generally given the opportunity to obtain necessary services, they face many barriers in accessing and utilising these services. These barriers include lack of information about or awareness of the services, insufficient supports to access these services and discordant expectations between the women and their service providers.

**Systematic review registration:**

PROSPERO registration number: CRD42012002185.

**Electronic supplementary material:**

The online version of this article (doi:10.1186/2046-4053-4-13) contains supplementary material, which is available to authorized users.

## Background

### Health equity is a priority for a multicultural Canada

Canada is currently experiencing large-scale immigration and increasing ethnocultural diversity [1]. Indeed, population growth over the past 100 years has largely resulted from immigration. Members of visible minority groups are expected to constitute between 29% and 32% of Canada’s population by 2031 [2]. The Canadian Charter of Rights and Freedoms not only affirms the multicultural nature of Canadian society but arguably also mandates equity in healthcare access and health outcomes for all Canadians, regardless of their place of birth [3].

As with newcomers in many other immigrant-receiving nations, the immigrants who enter Canada are relatively healthy. However, their health status converges towards the national average within 10 years of their arrival. A number of explanations have been suggested for this ‘healthy immigrant effect’ and its gradual erosion [4, 5], including initial selection of healthy individuals [4] and later acculturation; the stress of relocation, which may erode any initial health advantage [5]; and a distrust of western medicine and a preference for seeking out traditional healthcare options, which may result in poorer outcomes.

Unfortunately, the needs and rights of immigrant women are often marginalised by cultural practices within families and communities and sometimes by legislation. Socioeconomic marginalisation and the subsequent vulnerability of immigrant women can be further exacerbated by pregnancy and childbirth, making maternity an important focus of attention for those concerned with enhancing immigrant health.

### Perinatal health measures for immigrant women need revisiting

Epidemiological studies from Canada and elsewhere have reported equal or more favourable birth outcomes for migrants [6–9], thus supporting an ‘epidemiological paradox’ associated with the healthy immigrant effect. These results may apply specifically to immigrants from non-industrialised countries and may be associated with protective individual characteristics. Conversely, numerous other reports highlight serious problems of equity in birth outcomes [10–12], particularly for refugees [13] and other immigrants after increased lengths of stay [14, 15]. A systematic review in immigrant-receiving countries in Europe found substantial disadvantages for immigrants as compared to native-born women in all of their outcomes: their overall risks were 43% higher for low birth weight, 24% higher for pre-term delivery, 50% higher for perinatal mortality and 61% higher for congenital malformations [9]. Similarly, a recent Canadian study found higher rates of low birth weight and full-term low birth weight (that is, small for gestational age) for infants born to recent immigrant women [10]. Other negative newborn and maternal outcomes have also been observed, such as higher rates of gestational diabetes (predisposing the mothers to pre-eclampsia and type 2 diabetes and their offspring to obesity and type 2 diabetes) [16, 17]; low maternal weight gain (compromising both newborn and maternal health) [13]; genetic anomalies such as neural-tube defects due to lack of folic acid intake [18]; and maternal anaemia (increasing the risk of pre-term delivery) [19]. Any of these outcomes can affect maternal and infant health and well-being over their entire lifetimes. Because immigrant women often adhere to enduring traditional beliefs and practices despite their new environment, providing appropriate maternity care successfully will require the legitimization and incorporation of these beliefs and practices wherever possible [20, 21].

### Healthcare services are not appropriately utilised by immigrant women

Conflicting evidence exists regarding the under- or over-utilisation of health services by immigrant communities [22]. Some literature reports that women may have more frequent contact with health services than men due to maternity needs [5]. Alternatively, it has been found that many migrant women do not utilise formal health care and other community services, largely because of language barriers, difficulties in understanding healthcare information, experiences of discrimination and the challenges of navigating the Canadian healthcare system [23, 24]. Few explanations focus on the socioeconomic position of the immigrants, including material disadvantage, geography, racial harassment and exclusion [25–27].

Immigrant women often have difficulty in navigating the healthcare system during the prenatal, intrapartum and postnatal periods. These women may choose obstetric rather than midwifery care [28] or may opt for traditional services of their own background. Non-medical support for these women may be important to help them navigate the healthcare system and to access care during the postnatal period. In some areas, a doula (a non-medical labour coach) will provide important emotional support to immigrant women; these doulas are often unregistered midwives of immigrant backgrounds themselves. Recent research has found that migrant women often do not follow up on referrals for the post-birth care suggested by community health nurses [29], and thus any postnatal health concerns of these women may not be addressed by the healthcare systems in Canada [30]. Moreover, exposure to western biomedicine may powerfully influence immigrant women’s perceptions of maternal care, but these perceptions may not be congruent with their frames of reference. Similar challenges for newcomer women are documented in the international literature arising from Europe [31–33], Australia [34–36] and the United States [37, 38].

## Methods

### Study aim and objectives

The aim of this study is to provide stakeholders with perspectives on maternity-care services as experienced by immigrant women. We performed a systematic review using a narrative synthesis of findings from reports of empirical research. For the study, we employed ‘integrated knowledge translation’ (IKT), which has been described by the Canadian Institutes of Health Research (CIHR) as knowledge translation (KT) woven into the research process [39]. IKT requires partnering with key stakeholders (integrated knowledge users or IKUs) to ensure topic relevancy and to enable tailoring of messages and recommendations, which in turn facilitates effective end-of-study KT for application in future policy, practice or programming.

### Research question

Our research question was the following: What are the experiences of immigrant women in Canada in accessing and navigating maternity and healthcare services from conception to 6 months postpartum? Our focus was on (*a*) the accessibility and acceptability of maternity-care and related services for immigrant women and (*b*) the effects of the perceptions and experiences of these women on their birth and postnatal outcomes.

### Population of interest

We reviewed empirical and grey literature and other documents that report on immigrants in Canada, defining an immigrant as a person who has settled permanently in Canada [40]. This definition includes economic migrants, skilled workers, temporary foreign workers, documented and undocumented residents, refugee claimants, refugees, asylum seekers and students [41].

### Study design

Our systematic review employed narrative synthesis to identify, appraise and synthesise reports on empirical research. We reviewed studies with all types of designs: qualitative, quantitative or mixed-method. Narrative synthesis methods of systematic review (*a*) facilitate understanding and acknowledgement of the broader influences of theoretical and contextual variables, such as race, gender, socioeconomic status and geographical location; (*b*) enable understanding of the shaping of differences between reported outcomes as a result of differing study designs and childbearing populations; and (*c*) provide results that enable the development and implementation of maternity services and health interventions across diverse settings.

We used the narrative synthesis approach described by Popay et al. ([42]; p. 5), which is defined as ‘an approach to the systematic review and synthesis of findings from multiple studies that relies primarily on the use of words and text to summarise and explain the findings of the synthesis.’ This approach is equally suitable for both quantitative and qualitative studies, as the emphasis is on an interpretive synthesis of the narrative findings of research rather than on a meta-analysis of the data. Narrative synthesis allowed us to encompass cross-disciplinary and methodologically pluralistic research to document the experiences and outcomes of immigrant women in maternity. The major findings of this narrative synthesis are then used to explain how and why maternity services have been implemented and how these implementations have affected immigrant women of childbearing age.

### Search strategies and selection of studies

An information scientist (a health research librarian) designed the database search strategies, which in turn were reviewed by the entire research team (including IKUs) before implementation. The following databases were searched: Ovid MEDLINE In-Process and Other Non-Indexed Citations, Ovid MEDLINE Daily, Ovid MEDLINE (1950–2013), Ovid PsycINFO (1987 to present), Ovid EMBASE (1980–2013), EBSCOhost CINAHL (1937–2013), ISI Web of Knowledge Social Sciences Citation Index (1898–2013), ISI Web of Knowledge Science Citation Index (1899–2013), Scopus (1960–2013) and CSA Sociological Abstracts (1952–2013). We also performed hand searches within the websites of relevant journals such as *Journal of Immigrant and Minority Health; Journal of Obstetric, Gynecologic and Neonatal Nursing; Journal of Health Services Research & Policy; Canadian Journal of Public Health;* and *Culture, Health and Sexuality*. Team members also received training by the information scientist on search strategies for grey literature.

### Study selection

We employed a three-stage process: (*a*) screening; (*b*) preliminary categorization; and (*c*) retrieval, final selection and final categorization. In the first stage (completed for the database search but not for the grey literature search), one reviewer screened all citations retrieved from the database searches by applying a screening criteria checklist (Additional file 1). For a publication to be accepted, the first five criteria and one of the last two criteria had to be met to allow classification as ‘yes, empirical’ (*n* = 63) or ‘yes, non-empirical’ (*n* = 40). The ‘yes, empirical’ category was used for the narrative synthesis (systematic review) and the ‘yes, non-empirical’ category for a review of non-empirical literature. Literature that could not be confirmed as meeting the screening criteria were placed in a ‘maybe’ folder (*n* = 65), retrieved in full, and brought to one of the team leads (GH or MM) for a final decision.

The search for grey literature is including select database searches (ProQuest Dissertations and Theses, Google, and Google Scholar), internet-based searches (see Additional file 2), review of reference lists and email or phone contact with research and other stakeholders who have subject expertise or interest. Grey literature items were screened for relevance and were rejected if considered to be not of sufficient importance (categories 1, 2 and 3 in Table 1). These categories of grey literature were established for the fields of health services research and health policy by the National Information Center on Health Services Research and Health Care Technology at the National Library of Medicine [43]. Grey literature includes empirical research (using qualitative, quantitative or mixed-methods research) derived from the database searches (largely published in peer-reviewed journals) was placed in the narrative synthesis after confirmation of their empirical status (primary research using working hypotheses or research questions). All non-empirical publications were used for background or contextual information. The process of selecting grey literature and quality checks is detained in the protocol that published elsewhere [44].

**Table 1:** **Relative importance of grey literature as used by ICHSR and HCT at the National Library of Medicine** [48]

5	4	3	2	1
Working papers	Data evaluations	Speeches	Newsletters	Pamphlets
Committee reports	Foundation reports	Annual reports	Biographies	Protocols
Testimony	Government reports	Presentations	Bulletins	Guidelines
Conference proceedings	Grantee publications	Grantee reports	Slide presentations	Poster sessions
	Non-commercially published conference papers			
	Reports	Webcasts	Foundation financial statements	Meeting agendas
	Special reports	Theses		Translations
		Technical specifications and standards		

Grey literature also provided another dimension of identifying the gaps in the empirical research literature. The importance of reviewing this work partly stems from the fact that some non-empirical reports published in peer-reviewed journals, such as correspondence pieces, can highlight unique aspects of the topic that are not empirically studied, owing to, for instance, sufficient sample population (as with patient safety incidents, for example). It also relates to the fact that some audiences, such as policymakers and foundations, place a high priority on grey literature to gather their information [43].

Concurrently, the entire team engaged in preliminary categorization of the screened articles, the grey literature and the results of the hand searches. Two investigators worked independently in the subsequent final selection and categorization stage, with any disagreement being resolved by one of the study leads (GH or MM). Quality assessments of inter-rater reliability were performed within the narrative synthesis framework as described previously [44]. Please see Additional file 3 for our PRISMA flow chart and the reasons for exclusion.

### Data extraction and quality assessment

It is important to ensure the robustness of the synthesis is the methodological quality of key literature and the analytical methods used to develop the narrative synthesis. Research studies were critically appraised using the Joanna Briggs Institute (JBI) [45], Critical Skills Appraisal Programme (CASP) [46] and Crombie tools for survey [47]. We developed a weighting system—high, medium and low—using the criterion below in a previous study [48].

### Criterion statement

High—A study with a rigorous and robust scientific approach which largely meets all JBI benchmarks—perhaps 7 or more.

Medium—A study with some flaws but not seriously undermining the quality and scientific value of the research conducted—perhaps 5–7.

Low—A study with serious or fatal flaws and poor scientific value—perhaps below 5 of the benchmarks.

We considered the use of a weight-of-evidence approach such as that described by Gough [49]. Such an approach may not always be appropriate, however, especially in situations where insufficient information is available about the methodological quality of studies included in the review [48]. These procedures were performed to ensure that findings of the selected studies were credible and provided adequate level of understanding regarding maternity-care services in Canada. In addition, the knowledge gained from these studies could be transferable to the target audience [48].

## Results

We found 1,897 hits with 410 duplicates in our searches of the databases listed above. Additional file 4 contains further information regarding the search strategy used for Ovid MEDLINE. We proceeded to assess 68 articles for eligibility, including three grey literature and two hand-searched articles. A total of 24 articles (10 qualitative and 14 quantitative, see Table 2) were selected for the final study.

**Table 2:** **Study characteristics of all included articles (qualitative and quantitative)**

Author, Pub year	Study aim	Methodology qualitative studies	Sample characteristics	Key outcomes/findings	Quality tool used and appraisal
*Study characteristics of qualitative studies*					
1. Ahmed et al., 2008 [70]	Refugee, asylum seeking, non-refugee and immigrant new mothers with depressive symptoms were interviewed in a qualitative study to better understand: (a) their experiences and attributions of depressive symptoms; (b) their experiences with healthcare providers and support services; (c) factors that facilitated or hindered help seeking; (d) factors that aided recovery; and (e) factors which were associated with women continuing to experience symptoms of depression.	Semi-structured telephone interviews which were taped, transcribed, and analysed using a constant comparative approach.	10 immigrant mothers in Toronto, Ontario, who scored 10 or over on the Edinburgh Postpartum Depression Scale 7–10 days after giving birth, participated 12–18 months later. Two women had emigrated from China, 2 from India, 1 from Pakistan, 3 from South America, 1 from Egypt, and 1 from Haiti.	Many women attributed their depressive symptoms to social isolation, physical changes, feeling overwhelmed and financial worries. They also had poor knowledge of community services. Barriers to care included stigma, embarrassment, language, fear of being labelled an unfit mother and the attitude of some staff. Facilitators to recovery included social support from friends, partners and family, community support groups, ‘getting out of the house’ and personal psychological adjustment. Personal and systematic barriers exist in new immigrant mothers obtaining care for symptoms of depression.	Joanna Briggs Institute
					Low
2. Morrow et al., 2008 [64]	The study aimed to examine: (a) women’s experiences of depression after childbirth as described by the women themselves; (b) variables associated with psychosocial stress identified by the women as contributing to the experience of depression after childbirth; (c) the role of women’s family and community in the postpartum period; (d) the kinds of support sought by women in the postpartum period.	Ethnographic narrative approach utilising semi-structured, open-ended interviews.	18 first-generation immigrant women in Vancouver, British Columbia (7 Mandarin-speaking women, 8 Cantonese-speaking women, 3 Punjabi-speaking women) and 1 second-generation Punjabi-speaking immigrant woman.	The critical importance of the sociocultural context of childbirth in understanding postpartum depression suggests that an examination of women's narratives about their experiences of postpartum depression can broaden the understanding of the kinds of perinatal supports women need beyond healthcare provision and yet can also usefully inform the practice of healthcare professionals.	Joanna Briggs Institute
					Med–High
3. Reitmanova and Gustafson, 2008 [24]	The study aimed to document and explore the maternity healthcare needs and barriers to accessing maternity health services from the perspective of immigrant Muslim women.	In-depth semi-structured interviews.	6 immigrant Muslim women in St. John’s, Newfoundland.	Women experienced discrimination, insensitivity and lack of knowledge about their religious and cultural practices. Health information was limited or lacked the cultural and religious specificity to meet their needs during pregnancy, labour and delivery and postpartum phases. There were also significant gaps between existing maternity health services and women’s needs for emotional support and culturally and linguistically appropriate information. This gap was further complicated by the functional and cultural adjustments associated with immigration.	Joanna Briggs Institute
					Med
4. Spitzer, 2004 [66]	The study aimed to examine the relationships between nurses and visible (non-white) minority women giving birth in hospitals undergoing healthcare restructuring.	Interviews and focus group interviews using a semi-structured interview guide.	19 new mothers who had given birth in an unnamed Canadian province (5 First Nations, 6 South Asian Canadian, 5 Vietnamese Canadian, and 3 Euro Canadian). Also, 11 obstetrical nurses (4 foreign born and 7 Canadian born).	Nurses felt compelled to avoid interactions with patients deemed too costly in terms of time. Overwhelmingly, these patients were members of culturally marginalised populations whose bodies were read by nurses as potentially problematic and time consuming. As their calls for assistance went unanswered, visible minority women complained of feeling invisible. Taken in the context of historical and contemporary interethnic relations, these women regarded such avoidance patterns as evidence of racism.	Joanna Briggs Institute
					Low
5. Sutton et al., 2007 [60]	Vietnamese women’s breastfeeding experiences and challenges were explored, as were their families’ needs for prenatal and postpartum health professional programs and services.	In-depth, semi-structured interviews.	11 Vietnamese mothers of children younger than 2 years living in Middlesex—London, Ontario.	Lack of knowledge and misinformation were major barriers to breastfeeding. Inability to communicate in English and a lack of effective transportation were key obstacles to the women’s ability to access mainstream prenatal and postpartum health programs and services. Standard nursing prenatal and postpartum services appear not to have reached this group of mothers effectively.	Joanna Briggs Institute
					Med
6. Grewal et al., 2008 [18]	The study aimed to describe new immigrant Punjabi women’s perinatal experiences and the ways that traditional beliefs and practices are legitimised and incorporated into the Canadian healthcare context.	Naturalistic qualitative descriptive and focus groups.	15 first-time mothers who had immigrated in the past 5 years to Canada from Punjab, India and had given birth to a healthy infant in the past 3 months in a large urban centre in British Columbia, Canada; 5 health professionals and community leaders also took part in a focus group.	3 major categories emerged including: the pervasiveness of traditional health beliefs and practices related to the perinatal period (e.g., diet, lifestyle, and rituals); the important role of family members in supporting women during the perinatal experiences; and the positive and negative interactions women had with health professionals in the Canadian healthcare system.	Joanna Briggs Institute
					Med–High
7. Merry et al., 2011 [71]	The study aimed to gain greater understanding of the barriers that vulnerable migrant women face in accessing health and social services postpartum.	Qualitative text data on services that claimant women received post-birth and notes (recorded by research nurses) about their experiences in accessing and receiving services were examined. Thematic analysis was conducted to identify common themes related to access barriers.	112 asylum seekers/refugee claimants in Canada. 51 in Montreal, mainly from Nigeria, Mexico and India. 61 participants in Toronto, mainly from Nigeria, Mexico, Colombia and St. Vincent.	Of particular concern were the refusal of care for infants of mothers covered under IFHP, maternal isolation and difficulty for public health nurses to reach women postpartum. Also problematic was the lack of assessment, support and referrals for psychosocial concerns.	Joanna Briggs Institute
					Low–Med
8. Gagnon et al., 2010 [29]	The study aimed to explore the inhibitors and facilitators of migrant women for following through with referrals for care.	Semi-structured interviews.	25 women in Montreal, Quebec. 12 were asylum-seekers, 7 non-refugee immigrants, 5 refugees, and 1 Canadian-born. The 25 were born in 1 of 16 different countries (4—Pakistan, 3 each—Bangladesh and Sri Lanka, 2 each—India and Columbia, 1 each—the remaining 11 countries).	Inhibitors included language barriers, transportation problems, scheduling appointments, absence of husband, absence of childcare, cold weather, perceived inappropriate referrals and cultural practice differences. Facilitators included choice of follow-up facilitator, appropriate services, empathetic professionals and early receipt of information.	Joanna Briggs Institute
					Med
9. Ardal et al., 2011 [62]	The study aimed to: (a) explore the experience of non-English speaking mothers with preterm, very low birth weight (VLBW) infants (1,500 g); and to (b) examine mothers’ assessment of a peer support programme matching them with linguistically and culturally similar parent buddies.	An exploratory, qualitative analysis based on grounded theory. In-depth interviews using semi-structured guide.	8 Spanish, Portuguese, Chinese and Tamil immigrant mothers in an urban Canadian teaching hospital.	Study mothers experienced intense role disequilibrium during the unanticipated crisis of preterm birth of a VLBW infant; situational crises owing to the high-tech NICU environment and their infant’s condition; and developmental crises with feelings of loss, guilt, helplessness and anxiety. Language barriers compounded the difficulties. Parent buddies helped non-English speaking mothers mobilise their strengths. Culture and language are important determinants of service satisfaction for non-English-speaking mothers. Linguistically congruent parent-to-parent matching increases access to service.	Joanna Briggs Institute
					Low–Med
10. Wiebe and Young, 2011 [67]	The study aimed to explore the parent (client/patient) perceptions of culturally congruent care within a tertiary neonatal intensive care unit based on interviews with culturally diverse families with hospitalised infants. Attempting to further develop a new conceptual approach called the ‘Culturally Congruent Care Puzzle”, by incorporating the client/parent perspective.	Exploratory qualitative approach, grounded in an emic perspective, using open, non-directed interviews as much as possible.	21 families of diverse cultural origins, who had an infant in the neonatal intensive care unit in Edmonton, AB.	Key themes that emerged as elements of culturally congruent care were: (a) a relationship of caring and trust between the provider and client, (b) respectful and appropriate communication, (c) having social and spiritual supports that were culturally responsive and accessible and (d) having a welcoming and flexible environment.	Joanna Briggs Institute
					Med
*Study characteristics of quantitative studies*					
1. Kingston et al., 2011 [55]	The study aimed to compare the maternity experiences of immigrant women (recent, <5 years and non-recent) with those of Canadian-born women.	Secondary analysis of Maternity Experiences Survey with multivariable logistic regression.	A stratified random sample of 6,421 women who had recently given birth was drawn from a sampling frame based on the 2006 Canadian Census of Population. The total weighted sample comprised 7.5% recent immigrants (<5 years), 16.3% non-recent immigrants (>5 years) and 76.2% Canadian-born women. Roughly 50% of the immigrants were born in Asia.	Immigrant women reported experiencing less physical abuse and stress, and they were less likely to smoke or consume alcohol during and after pregnancy. They were more likely to report high levels of postpartum depression symptoms and were less likely to have access to social support, to take folic acid before and during pregnancy (due to lack of information), to rate their own and their infant’s health as optimal and to place their infants on their backs for sleeping. Fewer attended prenatal classes or travelled to give birth. Recent and non-recent immigrant women also had different experiences, suggesting that duration of residence in Canada plays a role in immigrant women’s maternity experiences.	Crombie
					Med–High
2. Brar et al., 2009 [52]	The study aimed to assess the use of perinatal care services by newly immigrated South Asian women and Canadian-born women and to determine any perceived barriers to receiving care.	Telephone survey consisting mainly of closed-ended questions.	2 groups of women in Calgary, Alberta: 30 South Asian women who had immigrated within the last 3 years and 30 Canadian-born women of any ethnicity.	Most women believed they had received all necessary medical care. Language barriers were most commonly reported by South Asian women and were considered to be the most common barrier to receiving care.	Crombie
					Med–High
3. Sword et al., 2006 [69]	The study aimed to describe immigrant women’s postpartum health, service needs, access to services, and service use during the first 4 weeks following hospital discharge compared to women born in Canada.	Data were collected as part of a larger cross-sectional survey study. Self-administered questionnaires and structured telephone interviews.	1,250 culturally diverse women in Ontario, Canada. 31.4% were born outside of Canada.	Immigrant women were significantly more likely than Canadian-born women to have low family incomes, low social support, poorer health, possible postpartum depression, learning needs that were unmet in hospital and a need for financial assistance. However, they were less likely to be able to get financial aid, household help and reassurance/support. There were no differences between groups in ability to get care for health concerns.	Crombie
					Med–High
4. Katz and Gagnon, 2002 [63]	The study aimed to ascertain need for larger scale study on postpartum care for immigrants for whom health and/or social concerns have been identified.	A descriptive, cross-sectional design was used to gather data from hospital and community records.	22 immigrant women. Families were not recorded as receiving optimal care.	40%–100% concerns not recorded as being resolved and 30%–100% of families were not recorded as receiving optimal care.	Critical Appraisal Skills Programme modified cohort Med-High
5. Minde et al., 2001 [68]	The study aimed to examine the extent to which physicians and nurses use their first postnatal contact with women to determine their psychosocial strengths and problems.	Interactions were audio taped and analysed. Edinburgh Postnatal Depression Scale, the Symptom Checklist-90-Revised and the Working Model of the Child Interview (WMCI) also used.	42 consecutively born infants and their mothers in Montreal, Quebec.	Recent non-Western mothers overrepresented among insecurely attached mothers.	Crombie Med-High
6. Gagnon et al., 1997 [35]	The study aimed to compare an early postpartum discharge programme versus standard postpartum care.	A randomised controlled trial. Experimental intervention consisted of discharge 6–36 h postpartum with nursing care available by telephone or at home at 34–38 weeks’ gestation and at ≤48 h and at 3, 5, and 10 days postpartum. The control included a postpartum stay of 48–72 h and standard follow-up.	175 healthy women recruited at 32–38 weeks in Montreal, Quebec. 21.7% were recent immigrants.	Early postpartum discharge coupled with prenatal, postnatal and home contacts leads to no apparent disadvantage. The programme may yield benefits for some mothers and infants, as it enhanced perceived maternal competence in recent immigrants.	Critical Appraisal Skills Programme RCT
					High
7. Gagnon et al., 2007 [30]	The study aimed to determine whether women’s postnatal health concerns were addressed by the Canadian health system differentially based on migration status (refugee, refugee-claimant, immigrant and Canadian-born) or city of residence.	Questionnaires and data extracted for hospital records. Questionnaires included visual analogue scale (VAS) for pain, the Edinburgh Postnatal Depression Scale (EPDS), the Personal Resources Questionnaire (PRQ) and the Abuse Assessment Scaler (AAS).	341 women of diverse migration status from Toronto, Montreal and Vancouver.	Differences in care provision were identified, suggesting that women and their newborn infants living in the largest Canadian cities may require additional support in having their health and social concerns addressed.	Critical Appraisal Skills Programme modified cohort (cross-sectional study)
					High
8. Poole and Ting, 1995 [57]	The study aimed to examine the relationship between cultural backgrounds and hospital maternity care.	Two studies were conducted using semi-structured in-person interviews/questionnaires.	The first study was comprised of 27 Euro-Canadian and 24 Indo-Canadian women. The second was comprised of 33 Euro-Canadian and 24 Indo-Canadian women.	The first study demonstrated the effects for cultural background on psychosocial variables but not biomedical factors. The second study determined that Indo-Canadian women had learned fewer baby care and self-care procedures and that nurses believed them to be less likely to use the procedures they had learned.	Crombie
					Low–Med
9. Chalmers and Omer- Hashi, 2000 [53]	The study aimed to explore perceptions of perinatal care and previous experiences with genital circumcision in Somali women who had recently given birth in Ontario.	Close-ended format interviews.	432 immigrant Somali women in the greater Toronto region, Ontario, with previous female genital mutilation, who had given birth to a baby in Canada in the past 5 years.	Women’s needs are not always adequately met during their pregnancy and birth care, and they are often unsatisfied with clinical practice and quality of care.	Crombie
					Med–High
10. Loiselle et al., 2001 [56]	The study aimed to document mothers’ perceptions of breastfeeding information and support received from hospital and community-based health professionals in a multiethnic community.	Telephone survey.	108 ethnically-diverse first-time breastfeeding mothers at 3 weeks postpartum.	Professional support perceived as positive, despite many experts considering the practise less than optimal. Immigrants had lower prenatal class attendance. Immigrant mothers agreed more strongly that hospital staff helped them feel confident with breastfeeding. Significantly more immigrant women received a home visit. More immigrant mothers had their babies receive supplemental water or formula; received formula samples upon discharge; and had staff demonstrate how to express milk if needed. Community-care nurses were more often a source of information for immigrant mothers; more Canadian-born mothers received information from a specialist.	Crombie
					High
11. Chalmers and Omer-Hashi, 2002 [65]	The study aimed to gain information about the perceptions of women with previous female genital mutilation (FGM) of their recent care during pregnancy and birth, as well as of their earlier genital mutilation experience.	Close-ended format interviews.	432 immigrant Somali women in the greater Toronto region, Ontario, with previous female genital mutilation, who had given birth to a baby in Canada in the past 5 years.	Findings suggest that women are frequently treated in ways that are perceived to be harsh and even offensive to cultural values. Women are, however, also appreciative of the clinical care they receive. There is a need to modify knowledge about female genital mutilation as well as attitudes towards women who have experienced this practice during perinatal care. Less interventionist clinical care and increased sensitivity for cross-cultural practices together with more respectful treatment are needed.	Crombie
					High
12. Stewart et al., 2008 [58]	The study aimed to determine if postpartum depression (PPD) symptoms are more common in newcomer women than in Canadian-born women.	Interview-assisted questionnaires for depression, social support, interpersonal violence and demographic information. A PPD variable was created based on a score of ≥10 on the Edinburgh Postnatal Depression Scale (EPDS), and a logistic regression analysis for PPD was performed.	495 consented to participate and 341 received home visits. 4 groups of women (65 refugees, 94 nonrefugee immigrants, 109 asylum seekers and 73 Canadian-born women) speaking any of the study languages and consecutively giving birth: Montreal, Toronto and Vancouver. All born outside Canada were <5 years in Canada.	Immigrants, asylum seekers and refugees were significantly more likely than Canadian-born women to score ≥10 on the EPDS, with the regression model showing an increased risk (odds ratio) for refugee, immigrant and asylum-seeking women. Women with less prenatal care were also more likely to have an EPDS of ≥10. Newcomer women with EPDS scores of ≥10 had lower social support scores than Canadian-born women. Social support interventions should be tested for their ability to prevent or alleviate this risk.	Crombie
					Med–High
13. Wallace et al., 2004	This study had four objectives: (a) to gain information on the barriers, needs and experiences of the newly postpartum women of non-Canadian/culturally diverse backgrounds who use maternal newborn services in Calgary, specifically from the PLC (Peter Lougheed Hospital in NE Calgary); (b) to assess needs and to determine gaps in the current delivery model; (c) to determine conditions and/or services that would enhance the utilisation of perinatal education and prevention programs by ethnocultural communities; and (d) to provide recommendations for future changes in service delivery models that allow for culturally competent care.	Survey study with questionnaire in hospital and approximately 2 weeks later.	Sample was non-random, convenience sample of 65 women interviewed over 5 months in 2002. Nurses identified women who did not speak English as a first language and 2 research assistants speaking 8 languages approached the women. 12 ethnicities represented with largest groups being South Asian (44.6%), West Asian/Arab (18.5%) and Chinese (12.3%). Almost 60% moved to Canada within last 7 years; 22.4% within last 2 years.	Prenatal care—78% stated no cultural barriers to prenatal care, but those identified were gender (*n* = 3) and language barriers (*n* = 3). 61% stated that their first preference was for a female doctor. None stated that cultural practices were discouraged. Of those trying to find a doctor of their ethnicity (*n* = 28), 83% were successful.	Crombie
				Prenatal care and information—98.5% received prenatal care, and 88% stated that it is very important. Mean weeks gestation found out pregnant was 6.2 and weeks contacted doctor was 7.5; 7.9 mean weeks at first prenatal appointment. 85% said that the physician explained things such that they could be understood and were open to questions from patients to clarify issues/concerns.	Med–High
				Accessing prenatal care—largest barrier was that they could not speak English very well (26.8%). Other issues identified by 10% or more were doctor only spoke English, office was too far away, did not have a way to get to doctor’s office and transportation too expensive. Only 7.7% said that they could not get a female doctor; and only 3.8% said they did not think they would feel welcome at the office/clinic.	
				Information on pregnancy—most women spoke to family physician or OB/GYN for information. Many (30%–40%) spoke to mother, in-laws and friends. 20% visited emergency for information. Only 5.8% stated public health nurse. No midwives or doulas.	
				Importance of receiving information—most women stated very important for numerous sources of receiving information including doctors, in-laws, mother, sister and friends.	
				Topics discussed—topics discussed less than 50% of the time included early bird prenatal classes, prenatal classes, low birth weight, group B streptococcus, support postpartum, birth control, baby care/ child restraints, new born screening and sexuality. Only 13% attended prenatal classes and less than 5% attended antenatal community care, the diabetic clinic and best beginnings. Child’s father identified as a great support by 90.6% of women (much more than other people).	
				Services at hospital—48.3% stayed longer than 72 h in the hospital, only 1 was discharged within 24 h (study recruitment tended to selectively recruit longer stay women.) 78.3% were satisfied with admission/discharge procedures at hospital.	
				Information gained whilst in hospital—95% got advice about care of their baby. Over 75% got advice about birth room care, breastfeeding, where to get help once at home, how to care for yourself, public health nurse visits and immunizations.	
				Helpfulness of advice received in hospital—most advice seen as very helpful; advice about care for baby least ‘very helpful’ with 73.1%. Topics discussed with public health nurse were comprehensive, except with respect to other resources for new parents (46.3% stated yes). Most women (90%) felt hospital staff was sensitive to cultural/religious beliefs.	
				Awareness of services—75.5% aware of prenatal classes; 63% of early bird (free) classes; approx 60% aware of other services.	
				Awareness of additional resources—92.3% were aware of book ‘Here through Maternity’, related to pregnancy resources in Calgary; about 60% aware of other resources.	
				Use of available services—only 23% of sample responded to these questions; approximately half attended prenatal classes but author noted that these classes are only offered for those having their first baby (and 45.3% of sample was having first baby).	
				Use of additional resources—most women used ‘Here through Maternity’ book, other books and several used libraries.	
14. Jarvis et al., 2011 [54]	The study aimed to assess the adequacy of prenatal care and perinatal outcomes for uninsured pregnant women at two primary care centres in Canada.	A retrospective case comparison study. A modified Kotelchuck Index was used to assess adequacy of care.	71 uninsured women in Montreal within a multiethnic community (3 of these women were Canadian Citizens). 72 control subjects were randomly chosen from provincially insured women presenting for prenatal care during the same period.	The study found that uninsured women presented for prenatal care 13.6 weeks later and had fewer blood tests, ultrasound screenings, cervical swabs, pap tests, genetic screening and visits with the healthcare providers (even when controlling for late initiation of prenatal care). There was no difference in the number who had physical examinations, the gestational age, birth weight, number of vaginal deliveries, number of inductions, use of epidural analgesia or attendance at the postpartum visit. The majority of uninsured women were categorised as having inadequate prenatal care utilisation. There was also a significant difference in the adequacy of received services.	Critical Appraisal Skills Programme cohort
					High

### Narrative synthesis

The general framework for a narrative synthesis comprises four elements: (*1*) development of a theory of how the intervention works, why it works and for whom; (*2*) development of a preliminary synthesis of the findings of the included studies; (*3*) exploration of the relationships in the data; and (*4*) assessment of the robustness of the synthesis. These elements are not necessarily independent of each other, and the synthesis often takes an iterative approach. Within each element, a variety of tools and techniques may be used depending on the nature of the research evidence. Additional tools and techniques may be used where appropriate [45]. A detailed description of each element and of the searching and screening process was reported earlier [44].

#### Element 1: developing a theory

Theory development did not play a large role in this synthesis, because we aimed to explore the experiences of immigrant women rather than to implement any intervention with measurable endpoints and outcome measures. However, a preliminary framework of the maternity-care experiences and outcomes of immigrant women was used to interpret and understand our synthesis. Developing this framework was an iterative process involving multiple revisions as we worked through elements 2–4.

#### Element 2: developing a preliminary synthesis

The preliminary synthesis provided an initial description and a map of the results of all the included research studies. This initial synthesis was further evaluated by the entire team to identify contextual and methodological factors that may have influenced the published results. Interrogation of the preliminary synthesis facilitated the construction of explanations as to how and why maternity services may have been implemented or may have affected immigrant women of childbearing age in a particular manner. We carefully organised the results, compared these results with other literature and noted the preliminary patterns that emerged regarding both the women’s experiences of maternity services and the development and implementation of health services and healthcare interventions. The results were then documented in the form of textual descriptions that included descriptive paragraphs under the subject headings of ‘Setting,’ ‘Participants,’ ‘Aim,’ ‘Sampling and recruitment,’ ‘Method,’ ‘Analysis,’ ‘Results’ and ‘Thick’ or ‘Thin’ study.

The assessment of publications as ‘thick’ or ‘thin’ as described by Roen et al. [50] had been previously been adapted by our team. This assessment method, which itself drew on the work of Denzin et al. [51], may be applied to both quantitative and qualitative narrative findings because the emphasis is on textual analysis of the narrative findings. We suggest that ‘thick’ papers (*a*) offer greater explanatory insights into the outcome of interest; (*b*) provide a clear account of the process by which the findings were produced—including the sample, its selection and its size, with any limitations or bias noted—along with clear methods of analysis and adjustments for confounding in statistical studies; and (*c*) present a developed and plausible interpretation of the analysis based on the data presented. In contrast, thin papers (*a*) offer only limited insights; (*b*) lack a clear account of the process by which the findings were produced; and (*c*) present an underdeveloped and weak interpretation of the analysis based on the data presented. Table 1 contains textual descriptions of the selected articles.

#### Element 3: exploring relationships in the data

Patterns emerging from the textual descriptions and cross-literature comparisons allowed us to identify the factors that affect maternity interventions and the implementation of maternity services. These factors were synthesised into five main themes regarding barriers and enablers that shape maternity services for immigrant women (described in the ‘Conceptual or thematic analysis’ section below). Throughout the synthesis process, careful attention was paid to the heterogeneity of research methods, methodological approaches and population characteristics encompassed in the literature.

**Figure 1:**
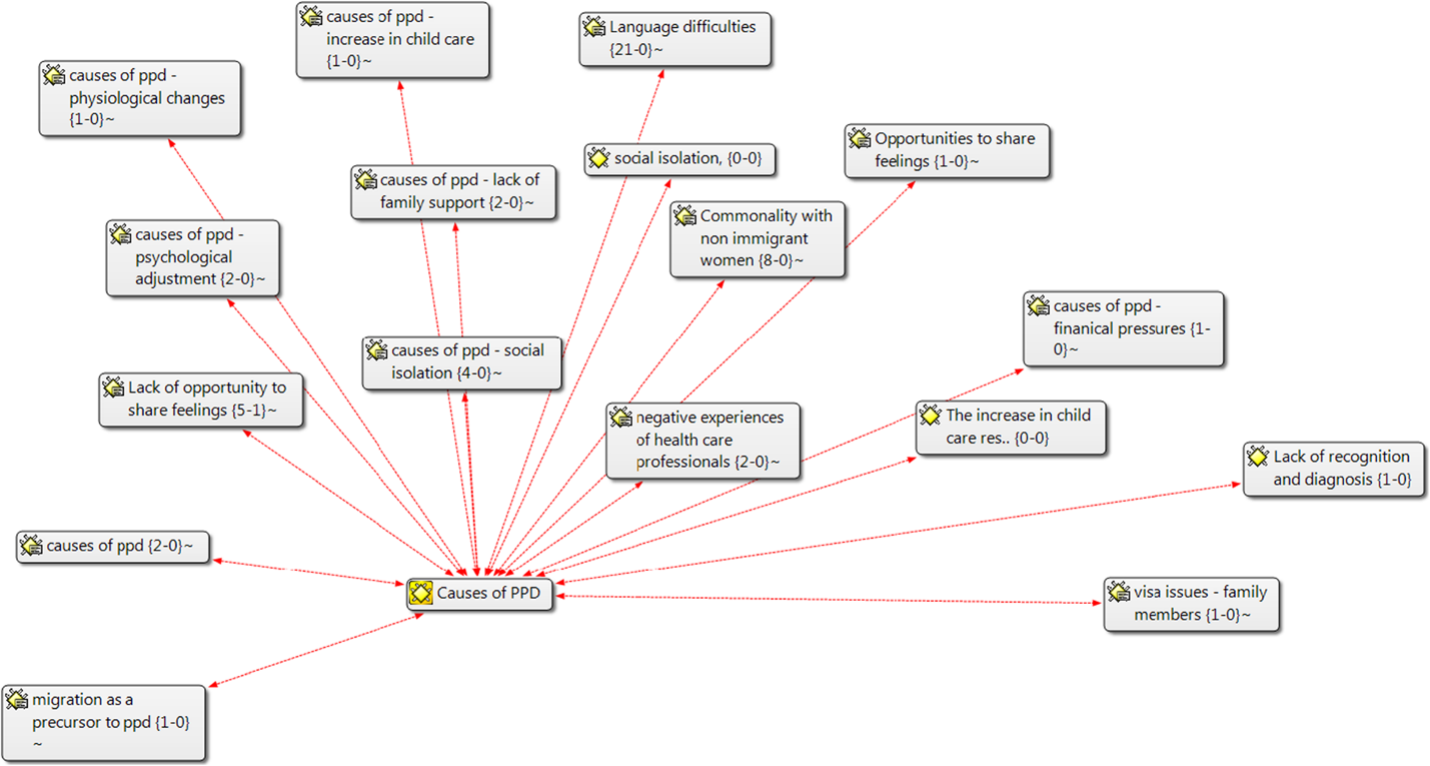
**Data analysis and themes—ATLAS.ti: example: causes of postpartum depression.**

**Figure 2:**
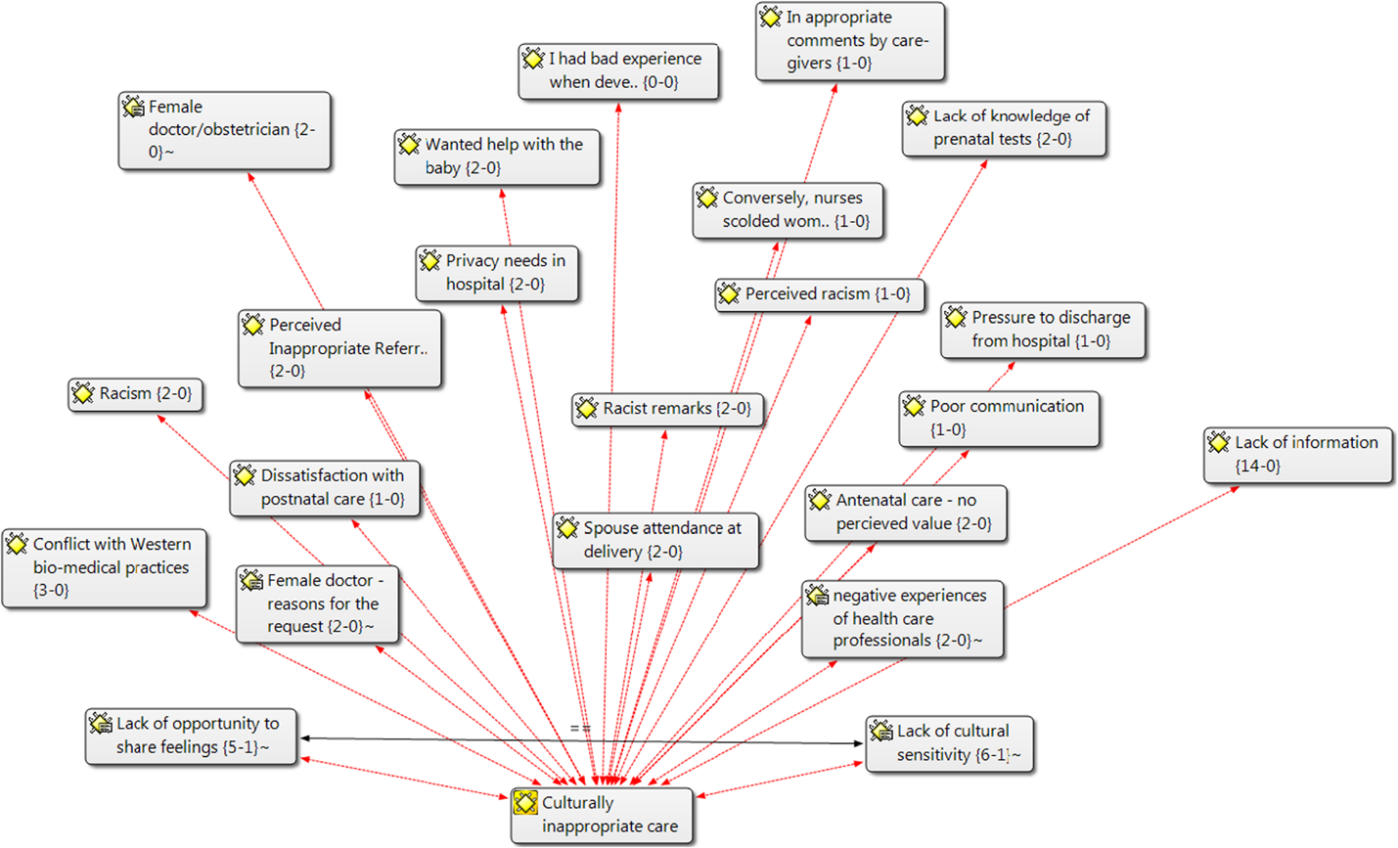
**Data analysis and themes—ATLAS.ti- example: culturally inappropriate care.**

In addition to tabulation, the findings were grouped and clustered using ATLAS.ti data analysis (see Figures 1 and 2) software (ATLAS.ti Scientific Software Development GmbH, Berlin, Germany) .The lead author has expertise in this software and consults as a trainer through an affiliation with the North American ATLAS.ti Training Center. Publications were uploaded as PDF files (Adobe Systems Inc., San Jose, CA, USA) and coded in accordance with the summary data requirements. The review team collectively decided on data variables (for example, study design and sample population), bearing in mind the categories that would be most informative for textual descriptions within the narrative synthesis. One reviewer extracted the data, and then one or more team members reviewed the data for accuracy and completeness. The publications selected for the non-empirical literature review were generally not research studies; hence, narrative descriptions of the key messages of those publications were constructed. Additional file 2 contains further information regarding data extraction.

#### Element 4: assessing the robustness of the synthesis

As mentioned earlier, the research studies appraised using the Joanna Briggs Institute [45], Critical Skills Appraisal Programme [46] and Crombie tools for survey [47]. We used a previously established weighting system—high, medium and low [48]. The quantitative studies were largely appraised as medium as high whereas the qualitative studies were largely medium and low quality in our appraisals. In team meetings, we discussed and documented potential sources of bias within the synthesis process, resolved discrepancies and built a consensus regarding the robustness of the synthesis. Our review of non-empirical literature provided additional understanding of the experiences that immigrant women have had with maternity-care services in Canada. This literature review was essential in part because some non-empirical reports (such as correspondence pieces published in peer-reviewed journals) can highlight unique aspects of the topic that are not empirically studied. In addition, some audiences (such as policymakers and foundations) use grey literature as a primary source of their information [43]. The literature review also identified concepts or themes reflecting the barriers and enablers that shape maternity services for immigrant women. Quantitative papers mainly addressed the issues and assessment of adequate or inadequate utilisation of perinatal care; satisfaction of prenatal care provided to the immigrant women; valuation of prenatal classes; assessment of postpartum depression Edinburgh Postnatal Depression Scale (EPDS) and breastfeeding and childcare issues. Qualitative papers largely described immigrant women’s experiences and concerns about their maternity care that includes education classes, breastfeeding difficulties, depressive symptoms, social isolation and lack of adequate support from the care services. The detailed description is narrated in the next section.

### Conceptual or thematic analysis

Searches of electronic databases and grey literature resulted in the selection of 24 primary research papers for thematic analysis, 14 of which used quantitative or mixed methods and ten of which were qualitative studies. The clinical contexts were heterogeneous: seven were perinatal, one prenatal, one prenatal and delivery, one delivery and 14 postpartum. Analysis of these 24 studies led to the development of five interrelated themes: (*a*) utilisation of prenatal care and educational classes; (*b*) adequacy of perinatal care; (*c*) barriers to maternity care in the pre- and postnatal periods; (*d*) isolation and limited social support; and (*e*) outcomes related to the access to and the use of services. The grid displaying these themes and their relationships between the studies is shown in Table 3. Immigrants’ population in these studies is from South Asia, West Asia and Arab countries; Europe and Vietnam.

**Table 3:** **The grid displaying these themes and their relationships between the studies**

Author, citation	Study design and broad category	Sample and ethnocultural group	Access to social support	Access/adequacy of prenatal care	Utilisation of prenatal care	Reception/ adequacy of care in hospital	Reception/adequacy of care – post- partum	Access to appropriate information	Barriers—prenatal	Barriers— postnatal	Postpartum health—mom (not mental health)	Baby health	Postpartum mental health	Breast feeding and other child care problems/success related to access
		Total	7	8	11	12	16	12	7	9	7	3	7	5
*Quantitative research papers*														
Kingston, JOGC, 2011 [55]	1. Secondary analysis of MES	1. Stratified random, 6,421 drawn from sampling frame	x		x	x	x	x			x		x	
	2. Maternity experiences	2. All but limited language (no Hindi or Punjabi)												
Brar, JOGC, 2009 [52]	1. Exploratory matched—sample survey	1. 30 immigrant, 30 Canadian		x	x	x		x	x					
	2. Experiences—use of perinatal services	2. South Asian												
Sword, JOGNN, 2006 [69]	1. Cross-sectional survey	1. 1,250 with 30% immigrants	x				x	x		x				
	2. Experiences and outcomes. Postpartum health, service needs, access and use	2. All but English, French, Spanish and Chinese languages												
Katz, CJNR, 2002 [63]	1. Retrospective cross-sectional hospital and community records review	1. 20 immigrant breastfeeding women with health or social concerns 2.9 countries	x			x	x	x			x	x	x	x
	2. Experiences and outcomes. Adequacy of postpartum care to 2 months													
Minde, JAACAP, 2001 [68]	1. Cross-sectional observational	1. 45 mother-infant pairs in Montreal with 45% immigrants					x				x (Psychosocial status more than maternal outcomes per se)		x	
	2. Experiences and outcomes. Nurse and physician adequacy of interviews	2. All, with English and French languages.												
Gagnon, Am J Ob Gyn, 1997 [35]	1.RCT	1. 54 treatment and 100 control				x	x					x		x
	2. Outcomes and experiences. Early postpartum discharge programme	2. Only 35 recent immigrants, had to speak English, French or Spanish												
Gagnon, CJPH, 2007 [30]	1. Matched cohort study with in-hospital questionnaire and data collected and in-person visits to home	1. 341 pairs of women from 10 hospitals					x			x	x		x	
	2. Experiences and outcomes. Unaddressed concerns in postpartum period (7–10 days after discharge)	2. if spoke 1 of 13 languages [Arabic, Dari/Persian, English, French, Mandarin/Cantonese (oral; ‘simple’ and ‘complex’ Chinese written), Punjabi, Russian, Serbo-Croatian, Somali, Spanish, Tamil and Urdu												
Poole, J Social Psychol, 1995 [57]	1. 2 observational studies with Euro and Indo-Canadian women in early postpartum period whilst in hospital	1. 27 and 24 and 33 and 24 Euro versus Indo-Canadian women			x	x								
	2. Experiences in hospital	2. Euro- and Indo-Canadians. Euro-Canadians all born in Canada and Indo-Canadians all born in India												
Chalmers, Birth, 2000 [53]	1. Survey study of Somalian women who had experienced FGM	1. 432 recruited within greater Toronto area in community and through snowball			x	x								x
	2. Perinatal experiences although some outcomes	2. Somalian												
Loiselle, CJNR, 2001 [56]	1. Cross-sectional survey study using telephone questionnaires at 3 weeks postpartum	1. 108 women with 69 being born outside Canada and 50% immigrants new <5 years, all living in Montreal			x	x	x	x						
	2. Experiences related to breastfeeding support	2. 30 countries but most prevalent countries being Philippines, Romania, Sri Lanka and Vietnam												
Chalmers, J Reprod Infant Psych, 2002 [65]	1. Mixed method study with closed and open-ended questionnaire. Report is based on open-ended questions which used ‘descriptive content analysis approach with quantitative methodologies’	1. 432 women of Somalian origin recruited from community sites and through snow balling				x								
	2. Experiences	2. Somalian												
Stewart, Can J Psych, 2008 [58]	1. Quantitative cross-sectional survey study with some collection from records.	1. Consecutive sample of 277 women in 4 groups recruited in 10 hospitals in Vancouver, Montreal and Toronto.											x	
	2. Outcomes via access	2. All with questionnaire translated into 13 languages												
Wallace, Calgary Health Region, 2002 [59]	1. A survey study with questionnaire in hospital and approximately 2 weeks later	1. Convenience sample of 65 non-English-speaking women. Almost 60% moved to Canada within last 7 years; 22.4% within last 2 years.		x	x	x		x	x					
	2. Experiences	2. 12 ethnicities represented with largest groups being South Asian (44.6%), West Asian/Arab (18.5%) and Chinese (12.3%).												
Jarvis, JOGC, 2011 [54]	1. Quantitative retrospective case comparison study was performed using medical charts	1. 71 uninsured women and 72 insured women presenting for prenatal care between 2004 and 2007 to 2 family practice centres in Montreal		x	x				x			x		
	2. Experiences (prenatal) and birth outcomes	2. All												
*Qualitative research papers*														
Ahmed, Arch Women’s Ment Health, 2008 [70]	1. Qualitative study using semi-structured telephone interviews 12–15 months after birth	1. 10 women, who scored 10 or over on EPDS at 2–3 week postnatal visit -Refugee, asylum seeking, non-refugee, and immigrants living in Toronto for less than 5 years	x				x	x		x				
	2. Experiences	2. 2 women had emigrated from China, 2 from India, 1 from Pakistan, 3 from South America, 1 from Egypt and 1 from Haiti.												
Ardal, Neonatal Networks, 2011	1. Exploratory, qualitative design based on grounded theory	1. Convenience. 8 non-English speaking mothers recruited from a Canadian NICU who had given birth to VLBW infants					x	x						
	2. Experiences	2. Spanish, Portuguese, Chinese and Tamil												
Gagnon, Journal of Immigrant Minority Health, 2010 [29]	1. Qualitative with individual and group interviews	1. 25 women were a combination of asylum seekers, non-refugee immigrants, refugees and Canadian born (*n* = 1)					x			x	x			
	1. Experiences	2. 16 different countries												
Grewal, JOGNN, 2008 [18]	1. Naturalistic, descriptive. Individual interviews with mothers. Focus group undertaken with Punjabi healthcare professionals to affirm results and offer recommendations.	1. 15 first time mothers, immigrated to Canada within the last 5 years from Punjab, given birth to a healthy infant in the last 3 months. Recruited from large BC hospital. 5 healthcare professionals were also recruited and took part in a focus group.		x	x	x	x		x					
	2. Experiences—the interaction with the Canadian healthcare system during the perinatal period	2. Punjabi women												
Merry, Qual Res, 2011 [71]	1. Subproject of prospective cohort study, with qualitative analysis of notes made by nurses and care diaries of women about refugee and newcomers’ services received	1. 112 research records of refugee women, who have been in Canada 5 or less years, claiming to have 3 or more unaddressed concerns, at either 2 weeks or 4 months post-birth, were reviewed (51 Montreal; 61 Toronto). Recruitment at 12 hospitals.	x				x	x		x	x		x	
	2. Experiences and outcomes postpartum	2. Montreal participants were mainly from Nigeria, Mexico and India; Toronto participants were from Nigeria, Mexico, Colombia and St. Vincent												
Morrow, Health Care Women Inter, 2008 [64]	1. Ethnographic narrative approach	1. 18 first-generation immigrant women (but most not newly immigrated), first language not English (Punjabi speaking, Cantonese speaking and Mandarin speaking), 1 year postpartum, either diagnosis of postpartum, or self-identified as having depression, post-birth.	x	x			x	x		x			x	
	2. Experiences postpartum (supports sought)													
Reitmanova, Matern Health Child J, 2008 [24]	1. Qualitative with in-depth interviews	1. 6 immigrant Muslim women in Newfoundland, born in 5 countries	x	x	x	x	x	x	x (in hospital)					
	2. Experiences—healthcare needs and barriers	2. Just reference to religion and ‘5 countries’												
Spitzer, Medical Anthropology Quarterly, 2004 [66]	1. Qualitative with individual and focus group interviews (not defined by number)	1. 19 new mothers who had given birth in 1 of 3 participating community centres or hospitals; also 11 obstetrical nurses were also interviewed about their experiences working with visible minority women				x			x (and in hospital)					
	2. Experiences in hospital	2. South Asian and Vietnamese (also First Nations)												
Sutton, Can J Diet Pract Res, 2007 [60]	1. Qualitative study with in-depth, semi-structured interviews	1. Heterogeneous sample of 11 (10 immigrant) Vietnamese mothers, whose children are under 2 years old		x	x		x	x	x	x				x
	2. Experiences with breastfeeding and needs for maternity services	2. Vietnamese												
Wiebe, J Transcult Nurs, 2011 [67]	1. Exploratory qualitative approach, grounded in an emic perspective, using open, non-directed interviews as much as possible	1. 21 families (13 immigrant and 6 Aboriginal) with infant in NICU	x			x				x (in hospital postnatal)				
	2. Experiences in a NICU	2. Several but largest African, Vietnamese and Eastern European												

#### Utilisation of prenatal care and educational classes

Research studies included in this systematic review generally reported that immigrant women were far less likely to have knowledge about or attend prenatal classes. Research findings from eight quantitative studies [52–59] and three qualitative studies [18, 24, 60] suggested that pregnant immigrant women receive less adequate perinatal care than pregnant non-immigrant women [24, 54–60]. Several studies reported that the two groups had similar rates and timings of seeking prenatal care by physicians, but found that immigrant women were far less likely to have knowledge about or attend prenatal classes [56, 57, 60]. A survey of South Asian immigrants in Calgary showed that 53% of respondents were unaware of prenatal classes [52]. Another study in same city but with a diverse sample reported high awareness (75%) of prenatal classes (but unknown utilisation levels) [59]. Several studies reported that the reasons for not attending prenatal classes were lack of transportation, language barriers [18, 57, 59] and negative perceptions about the type of information provided [18, 55]. Nonetheless, some immigrant women believed that prenatal classes and prenatal care were helpful because they had been fearful of the childbirth experience, particularly in the absence of any other support at home. In general, the studies recommended that prenatal education and classes should be offered in the language of the immigrants, with particular attention being given to the cultural and ethnic context [52, 54, 60]. The classes should also be offered in convenient locations and address topics of importance to the immigrant population [59].

Only a few studies reported on the breastfeeding information that immigrant women received from hospital staff [24, 54, 61]. Two studies showed that a lack of knowledge and information was a major barrier that prevented immigrant mothers from initiating breastfeeding [18, 61]. Some immigrant women reported that they were unable to start breastfeeding because they lacked peer support and knowledge about how to handle their babies [62]. Other immigrant women believed in the benefits and convenience of breastfeeding and had peer support to facilitate its use [24]. Data available from a Canadian census showed that more immigrant women than Canadian women received help to start breastfeeding in the hospital [55]. In another study, immigrant mothers reported being satisfied with the support for breastfeeding, but more immigrant mothers had their babies receive supplemental water or formula, received formula samples upon discharge and had staff show them how to express milk if needed [56]. However, studies have reported the intense level of difficulties faced by immigrant women after discharge without documentation of resolution of these issues [63]. It was also documented that recent immigrant women in Quebec were 2.2 times more likely to breastfeed [35]. These studies suggest that providing culturally relevant explanations about breastfeeding and other prenatal preparations are important and should be included in programs for pregnant immigrant women in Canada.

#### Adequacy of perinatal care

Although some immigrant mothers reported satisfaction with the adequacy of perinatal care they received, satisfaction was not consistent across the country. For clarity, we have subdivided this theme into three categories: (*a*) prenatal care [18, 24, 52, 54, 59, 60, 64]; (*b*) delivery and in-hospital care [18, 24, 52–57, 63, 65–67]; and (*c*) postpartum care [18, 24, 29, 30, 35, 55–57, 60–64, 68–71].

Some immigrant mothers reported inadequate utilisation of prenatal care because they lacked health insurance coverage; for example, the majority of uninsured women in Montreal were categorised as having inadequate prenatal care utilisation [54]. Some reports showed that immigrant mothers were sometimes not informed about the availability of prenatal classes, their purpose or the support offered to attend them. Reitmanova and Gustafson [24] qualitatively explored the maternity healthcare needs and the barriers to accessing maternity health services that existed for immigrant Muslim women living in St. John’s, NL, Canada. This study reported that some immigrant women were not told about these classes or did not understand their purpose; some did not attend the classes because the no-care arrangements were offered for their other children; and some refused to participate in these classes because they were not designed exclusively for women. Attending these classes together with men would have caused observant Muslims great discomfort because it would have contravened their religious beliefs [24]. In addition, immigrant women who exhibit symptoms of stress and anxiety in the prenatal period may have a high risk of postpartum depression. These women reported that their stressors included migration issues, language difficulties, marital conflict, conflict with family members and in-laws and physical health problems [59, 64].

Studies reporting on the adequacy of delivery and in-hospital care showed mixed perceptions among immigrant mothers. Some studies reported satisfaction for the care the mothers received in the hospital [54–56]. These studies provided positive accounts of the immigrant pregnant women receiving support for the promotion of breastfeeding during hospitalisation. Loiselle et al. [56] reported that immigrant mothers perceived that they were more likely to receive health professional support for breastfeeding than were Canadian-born mothers. Similarly, Wallace et al. [59] found that 90% of the pregnant immigrant women in their study thought that hospital staff were sensitive to their cultural and religious beliefs [59]. However, other studies reported some dissatisfaction. Muslim women in one study (*n* = 6) experienced remarks that were insulting, insensitive, stereotypical and embarrassing [24]. Somali women had several issues with their care, often related to their female genital circumcisions: (*a*) less than 1% had wanted a caesarean but 50% had received one; (*b*) some complained of having little discussion or say about procedures related to their birth and pain management; (*c*) 87.5% reported that hurtful comments had been made by their caregivers related to their circumcisions; and (*d*) many thought the nurses regarded them as being lazy (83.6%) or reluctant to cooperate (79.6%) [53]. Indo-Canadian immigrant women learned fewer baby care and self-care procedures than non-immigrant women [57]. Several Punjabi women reported experiencing difficulty during their hospital stay because they were not served hot food; hospital staff instead provided cold food such as sandwiches, Jell-O, and salads [18]. In addition, nurses are compelled to encourage women to begin walking and washing as soon as possible following delivery to enable their early discharge, which may conflict with cultural prescriptions of strict bed rest or the avoidance of showers [63]. Various studies reported incidents of poor treatment received from the hospital staff with respect to cultural sensitivity, delivery options, pain management and communication with immigrant mothers [52–56, 59, 63–67].

The adequacy of postpartum care experienced by immigrant mothers also varied greatly. Those in Ontario were reported to have good postpartum access to an obstetrician or public health nurse [69], but immigrant mothers in other parts of the country were not routinely contacted by a healthcare provider after discharge to address issues such maternal depression and social isolation [55, 63]. Kingston et al. [55] compared the maternity experiences of recent immigrants (5 years or less in Canada) and non-recent immigrants (more than 5 years) with those of Canadian-born women; after discharge, fewer recent immigrants than Canadian-born women were contacted by a healthcare provider (84% versus 95%) or saw one (70% versus 79%) other than in routine visits [55]. Similarly, in 12 of 20 immigrant mother-infant pairs, Katz and Gagnon [63] found no charted evidence either of contact within 48 h of discharge or of interventions to address maternal depression and social isolation.

The satisfaction that immigrant mothers feel with the perinatal care they receive from nurses, physicians and the healthcare system is evidently related to multiple factors. Consideration of these factors will be important in tailoring context-based programs to ensure the delivery of adequate maternity care to immigrant women.

#### Barriers to maternity care in the pre- and postnatal periods

Almost all the selected studies identified barriers in the delivery of maternity care to immigrant women in Canada, primarily involving access problems or negative experiences. These barriers include lack of knowledge among immigrant mothers, especially of knowledge and programs related to breastfeeding and postpartum depression [60, 70]; language barriers [60]; transportation problems, difficulties in making appointments, absence of partners, absence of child care, cold weather, perceptions of inappropriate referrals and differences in cultural practices [29]; wait times for appointments [64]; the presence of men in prenatal classes (in contravention of the religious beliefs of the women) [24]; lack of health insurance [24, 54]; concerns about the ability of the family to manage potential complications [66]; and time and money constraints [24, 52, 60].

Immigrant mothers in Montreal (speaking English or French) agreed more strongly than Canadian-born mothers that hospital staff helped them to feel confident in breastfeeding; Canadian-born women felt that they had received contradictory information from hospital staff [56]. In general, however, a lack of accurate information about breastfeeding was considered a major problem [60, 63]. Immigrant women reported that they found no community support related to breastfeeding and postpartum depression [60, 63, 64]. Immigrant women also said that they did not discuss their feelings of depression with their doctors or other healthcare professionals in their pre- or post-delivery check-ups because they felt too rushed or were not asked about possible emotional disturbances [70]. Lack of access to appropriate information and education related to family planning and immunisation were also reported as barriers to adequate maternity care [52, 55, 56, 63, 69].

Most studies highlighted issues related to the use of English in providing maternity care to immigrant women. For example, some immigrant women stated that language difficulties were the only barrier that would prevent them from using maternity services [70]. However, some immigrant women living in Vancouver (a large multi-ethnic urban centre) were fortunate to have access to healthcare professionals and resources in one of their preferred languages [56]. Studies have also discussed the attitudes of healthcare professionals towards immigrant women during and after hospitalisation. Several women reported being discouraged by staff who did not seem genuinely interested in overcoming the language barrier to help them or to provide appropriate information [63, 64, 70].

Cultural differences also caused difficulties for immigrant women in comprehending information, and some complained that maternity care is not always culturally and linguistically appropriate for them [24]. If these women failed to follow instructions because of their cultural preferences, healthcare staff often labelled the women as lazy or noncompliant [65]. Few immigrant women perceived that healthcare providers were insensitive to their pain or failed to provide them with special care when they were in need [65, 70]. Nonetheless, Katz and Gagnon [63] stated that 87.5% of women reported that hurtful comments were made by healthcare professionals at some stage of their maternity care.

The studies we reviewed indicated that immigrant mothers generally did not receive appropriate information regarding maternal and child health before and during their pregnancy [55] or after delivery of their babies [69]. In some instances, interpreter services were not available (as in Montreal), thus preventing some immigrant mothers from expressing their concerns or understanding the classes and information that were provided to them [71]. Mothers with postpartum depression who accessed psychological support received the information about this support primarily from friends or advertisements [70].

#### Isolation and inadequate social support

Immigrant parents identified social support as a key factor that helped them in accessing maternal services in Canada. However, immigrant women were more likely than Canadian-born women to perceive that they received less social support during their pregnancy or the postpartum period. Immigrant women appreciated and valued the limited social support they received from community services; but this support was not enough to overcome their problems and most of the time, they felt isolated and secluded [55, 69]. Immigrant parents with babies in neonatal intensive care units reported their appreciation of the social support they received (sometimes via long-distance telephone) from family, friends, staff, a spouse or partner, other children and their religious community [67]. However, immigrant women in other studies reported receiving only weak support during their pregnancy [24, 64, 69]. The women often expressed the wish to have someone from their community with previous maternity experience to spend some time with them, but this was not always possible [24]. Some immigrant women said that they did not know where to get maternity information, they felt isolated and they perceived that no one was available to help them and their babies [64, 63, 69, 71]. Sword et al. [69] reported that particularly after the birth of the newborn, immigrant women were less likely than Canadian-born women to get household help, reassurance and support or financial assistance; the main reason cited was a lack of information about the social support services available for the immigrants. Immigrant women also contrasted their experiences of social isolation with the situation in their countries of origin, which often placed high importance on family members and friends providing social support immediately after the birth of a child [65].

The types of community services available and the roles played by community health nurses were identified as crucial to the support received by immigrant mothers [64]. For new mothers of South Asian and Chinese origin who had postpartum depression and who described outside support as helpful, community health nurses typically played key roles [60]. Unfortunately, immigrant women generally received low social support at home even when psychosocial concerns were identified and documented [66, 69]. These mothers reported lack of knowledge with regard to the availability of support services in the community related to issues such as breastfeeding and postpartum depression, often leading to feelings of social isolation [60, 71]. Lack of social support and feelings of isolation have led to physiological problems documented in the hospital records of many immigrant women. For example, about 45% of the immigrant women had psychosocial concerns (largely anxiety and social isolation) identified in their hospital or community clinic charts [63]. Overall, lack of adequate social support and feelings of isolation were described in a variety of ways in three quantitative [55, 63, 69] and four qualitative [24, 67, 70, 71] studies.

#### Outcomes related to the access to and the use of services

Many of the selected studies described various outcomes of pregnancies, including birth outcomes [54, 67], postpartum mental health [30, 55, 58, 64, 69–71], and breastfeeding and childcare issues [35, 53, 60, 63, 65]. Many immigrant mothers mentioned that the low quality of maternal services available for them in their home countries, including unsanitary conditions and the requirement to pay, resulted in poor birth outcomes there [67]. However, some immigrant women had similar birth outcomes in their new country: uninsured immigrant women who received inadequate prenatal care were reported to suffer from poor outcomes with respect to gestational or foetal age, birth weight, number of vaginal deliveries, number of induced deliveries, the use of epidural analgesia and attendance at the postpartum visit [54].

The access to and use of postpartum mental health services by immigrant mothers were explored in several studies. One study showed that newcomer women with less prenatal care (beginning later in the pregnancy or limited in amount) were two times more likely to have an Edinburgh Postnatal Depression Scale score of greater than or equal to 10 (*P* = 0.03) [58]. In another study in Montreal of 50 refugee mothers who received a home visit at 4 months postpartum, 26 were found to have symptoms of postpartum depression and 16 reported skipping meals because of a lack of resources [71]. Almost none of the women with postpartum depression had sought out referrals to psychological support services via nurses: they had accessed the information through friends or advertisements instead [70]. Many immigrant women also reported that their interactions with healthcare practitioners were not supportive, but few described these encounters as critically important; the studies recommended that support for these women move beyond the medical management of depression [64, 65]. Several studies concluded that immigrant women require a range of postpartum supports that take into account social, cultural and other contextual factors [64, 68, 71].

Fewer immigrant women than Canadian-born women were reported to have accessed prenatal classes on breastfeeding and childcare; for example, in a study of 20 immigrant mothers via reviews of hospital and community clinic charts, eight were found to have breastfeeding difficulties. Resolution was recorded in two of these, referral was documented in one, six had been unable to resolve their problems in hospital, five still had problems on follow-up with a community nurse and one had already switched to bottle-feeding [63]. Despite fewer newcomers attending prenatal classes, breastfeeding initiation rates were found to be similar for immigrant and Canadian-born women, and immigrant mothers were 1.7 times more likely to be breastfeeding at 3 months [55].

## Discussion and recommendations

Our knowledge synthesis of maternity care among immigrants to Canada provides a coherent evidence base not only for understanding the factors that generate disparities in accessibility, acceptability and outcomes during maternity care but also for improving culturally based competency in Canadian maternity care. Our synthesis has identified pertinent issues that multiple sectors may use to configure maternity services and programs appropriately.

Problems related to the communication of appropriate information, the provision of culturally safe and appropriate care, the promotion of breastfeeding and access to care can be increased by providing appropriate multi-sectorial and multidisciplinary services. Social and other network services can work together with health professionals to ensure that immigrant women receive culturally congruent and culturally safe maternity care. Enhancements to maternity care for immigrant women will ultimately benefit not only these women but also the health of future generations of Canadians [53, 64, 70].

This narrative synthesis served as a tool for drawing together messages from empirical research to guide policy and practice in meaningful ways. The heterogeneous nature of the methodological approaches of the studies reviewed—quantitative, qualitative and mixed-method—militated against a meta-ethnographical approach or a meta-data analysis. However, the narrative synthesis clearly revealed that maternity care for immigrant women in the prenatal, perinatal and postnatal periods should focus not only on the real and perceived sources of emotional and practical support but also on contextual factors [62–71]. Our findings, carefully interpreted, will allow knowledge users within multiple sectors to strategically enhance maternity-care services and professional development to ensure timely and appropriate provision of culturally congruent maternity care. Recommendations and key messages drawn from the review are the following:

- Healthcare professionals should be aware not only of the basic postpartum health needs of immigrant women but also of their income, learning and social support needs (especially their community resources). Such awareness will help to ensure effective interventions and referral mechanisms, particularly for income and social support services. Referral pathways to cultural or faith-based health and social programs should be established or improved, and accessible healthcare information should be provided to migrants upon arrival at the border [29].
- Public health nurses should be trained to understand the cultural manifestations and cultural context of postpartum depression [69].
- More communication should be encouraged between nurses and physicians to allow better knowledge transfer and collaborative care, particularly for psychosocial issues [68].
- Although immigrant women in Canada are generally given the opportunity to obtain necessary services, they face barriers to accessing and using them. These barriers include not only the lack of availability or awareness of information and supports but also the presence of discordant expectations on the parts of the women and the service providers.

### Dissemination of findings

Our dissemination goals are twofold: 1) To ensure that key messages are delivered in an audience-specific manner, to optimise their effect on policy and practice change throughout the health service and the public health, immigration and community sectors; and 2) to utilise widely accessible technology (webinars and possibly social networking) to ensure maximum coverage. The specific KT strategies can be categorised by the target audience.

For mixed-audience KT, a research briefing paper for health practitioners, policymakers and decision-makers in Canada and an accessible plain-language fact sheet is professionally designed and produced, with wide dissemination using the activities and networks of academic team member and IKUs. The fact sheets, in particular, facilitate transfer of messages to the public and healthcare professionals, policymakers and other knowledge users. Contribution to academic theory and practice will occur through publication of findings in accessible international journals. The IKUs are also invited to co-author publications.

Given that the research team involves people who are engaged in community and hospital-based health services with immigrant women, knowledge translation has begun and will continue through to public dissemination through community meetings with families, women’s groups and workshops related to refugee/immigrant health. Likewise, additional knowledge users (multiprovincial) will be invited to attend community-based seminars/workshops. Publicising our findings at multicultural and immigration/multicultural events will target community, provincial and national leaders; they are the primary agents for these activities.

## Conclusions

This narrative synthesis has facilitated understanding and acknowledgement of the broader influences of theoretical and contextual variables in maternity care, such as race, gender, socioeconomic status and geographical location. It also enabled a better understanding of how differences between reported outcomes and study designs in relation to childbearing populations are shaped by the development and implementation of maternity services and health interventions across diverse settings. The systematic search and narrative synthesis highlights the overall perceptions and experiences of immigrant women on their birth and postnatal outcomes importance and problems related to accessibility and acceptability of maternity-care and related services for these women. However, country-specific origins not determined because of limited population from different parts of the world are included in the study. The additional review of non-empirical reports was used to supplement and contextualise the narrative synthesis findings and will enrich our understanding and dissemination of the findings.

## Electronic supplementary material

Additional file 1: **Screening criteria checklist.** (PDF 224 KB)

Additional file 2: **List of websites generated for searching grey literature.** (PDF 197 KB)

Additional file 3: **PRISMA 2009 Flow Diagram.** (PDF 90 KB)

Additional file 4: **Search Strategy overview and table for MEDLINE: immigrant women’s maternity-care experiences in Canada.** (PDF 81 KB)

## Abbreviations

ICHSR: Information Center on Health Services Research
HCT: Healthcare technology
IKT: Integrated knowledge translation
KT: Knowledge translation
IKU: Integrated knowledge users.
