# Supplementary material for: Immigrant women’s experiences of maternity-care services in Canada: a systematic review using a narrative synthesis
Source: Syst Rev. 2015 Feb 11;4:13. doi: 10.1186/2046-4053-4-13 (PMC4506414; doi:10.1186/2046-4053-4-13)
Supplement: Supplementary file 1 — Additional file 1: Screening criteria checklist. (PDF 224 KB) [file 13643_2014_343_MOESM1_ESM.pdf]

Additional file 1: Screening criteria checklist.

Item	Yes	No	Can't say
1. Publication date 1995-Dec 2011			
2. English or French language			
3. The paper discusses women living in Canada			
4. The paper discusses immigrant women or newcomers			
5. Is related to pregnancy and/or the perinatal/postnatal period?			
6. Is related to experiences and/or perceptions of maternity care/perinatal care?			
7. Discusses access and navigation of maternity care/perinatal care and/or pregnancy, perinatal or postnatal outcomes of immigrant and newcomer women?			

The first five items and one of the last two must be met for retention. Literature with 'can't say' checks are being placed in the 'maybe' folder for full retrieval and further screening
