# Supplementary material for: Immigrant women’s experiences of maternity-care services in Canada: a systematic review using a narrative synthesis
Source: Syst Rev. 2015 Feb 11;4:13. doi: 10.1186/2046-4053-4-13 (PMC4506414; doi:10.1186/2046-4053-4-13)
Supplement: Supplementary file 2 — Additional file 2: List of websites generated for searching grey literature. (PDF 197 KB) [file 13643_2014_343_MOESM2_ESM.pdf]

Additional file 2: List of websites generated for searching grey literature

During searches will identify search terms used and outcomes as relates to 1. Searched, nothing found, 2. Searched, relevant items found, 3. Not searched, 4. Searched items may be of relevance for background

Clinical practice guidelines sites

1. Canadian Medical Association. CMA Infobase. http://www.cma.ca/index.php/ci_id/54316/la_id/1.htm
2. Ontario Guidelines Advisory Committee. (GAC) http://www.gacguidelines.ca/index.cfm?pagepath=GAC_Endorsed_Guidelines&id=21080
3. British Columbia Ministry of Health Services http://www.bcguidelines.ca/gpac/alphabetical.html
4. Alberta Medical Association Towards Optimized Practice http://www.topalbertadoctors.org/

Women's health

1. Mother and Child health Research (Aus) www.latrobe.edu.au/mchr/
2. Centre for Research on Educational and Community Services: Ontario's Maternal, Newborn www.rrasp-phirn.ca
3. Best start http://www.beststart.org/ and http://www.beststart.org/index_eng.html and http://www.beststart.org/resources/rep_health/index.html
4. Health Canada's women's health pubs http://www.hc-sc.gc.ca/hl-vs/pubs/women-femmes/index-eng.php
5. The Source: Women's Health Data Directory: Provides links and annotations to data sources of women's health. Funded by Canadian federal and provincial government organizations: http://www.womenshealthdata.ca/advancedsearch/default.aspx?rt=2
6. http://www.womenandhealthcarereform.ca/
7. http://www.motherhoodinitiative.org/
8. Canadian women's studies assoc http://www.yorku.ca/cwsaacef/
9. http://thesurvey.womenshealthdata.ca/
   The Survey/Le Sondage is a bilingual web based repository that links women's health practitioners, policy makers, health authorities, and women's groups to a range of resources on women's health, including international, national, and provincial documents. Materials such as local health authority reports, reports commissioned by governments, and those produced by and for community groups and citizens are included. The Survey/Le Sondage allows users to search for specific reports and other sources of grey literature that are pertinent to both the geographic area and topic of interest.
10. Canadian women's health network http://www.cwhn.ca/en
11. Centres for excellence for women's health http://www.cewh-cesf.ca/en/index.shtml database http://www.cewh-cesf.ca/en/search.shtml and research bulletin http://www.cewh-cesf.ca/en/publications/RB/index.shtml
12. Canadian foundation for women's health http://www.cfwh.org/
13. SOGC Women's Health Information http://www.sogc.org/health/index_e.asp
14. Women's Health Research Foundation of Canada http://www.whrfcinc.com/
15. Women's college hospital http://www.wchospital.ca/

16. Women and health protection http://www.whp-apsf.ca/en/index.html
17. Ontario's women's health network
18. The Source: women's health data directory http://www.womenshealthdata.ca/resources/
19. Women's health research network http://www.whrn.ca/opportunities.html
20. Canadian association of women's health and perinatal nurses http://www.capwhn.ca/en/capwhn/Home_Page_p2469.html
21. Women's health in women's hands (Ontario health services site) http://www.whiwh.com/

Immigrant/multicultural women's health/associations

1. Immigrant Women's Health Centre (Toronto) http://immigranthealth.info/
2. Immigrant Women's Centre (Ontario) http://www.stjosephwomen.on.ca/
3. Migration and reproductive health http://migrationandreproductivehealth.org/?page_id=13
4. South Asian Women's Community Centre http://www.sawcc-ccfsa.ca/EN/
5. Indo-Canadian Women's Association http://www.icwaedmonton.com/
6. South Community Birth program http://scbp.ca/helpful-resources.html
7. Calgary Immigrant Women's Association http://www.ciwa-online.com/

Provincial/federal health care & policy sites

1. Status of Women of Canada http://www.swc-cfc.gc.ca/index-eng.html
2. Canada Prenatal Nutrition Program http://www.phac-aspc.gc.ca/hp-ps/dca-dea/prog-ini/cpnp-pcnp/about-apropos-eng.php
3. Canadian Public Health Association http://www.cpha.ca/en/default.aspx
4. Health Evidence http://health-evidence.ca/
5. Public Health Research, Education and Development http://www.phred-redsp.on.ca/
6. Health Canada – migrant health http://www.hc-sc.gc.ca/sr-sr/pubs/hpr-rpms/bull/2010-health-sante-migr/index-eng.php and health policy research bulletins http://www.hc-sc.gc.ca/sr-sr/pubs/hpr-rpms/index-eng.php
7. CHNET-WORKS Population Health Improvement Network http://www.chnet-works.ca/
8. Gov Canada Policy Horizons Canada (Policy research initiative) http://www.horizons.gc.ca/homepage.asp?pagenm=root&langcd=E
9. Canadian Association of midwives http://www.canadianmidwives.org/resources.html
10. Access alliance http://accessalliance.ca/content/endorse-right-health-care-coalition
11. Canadian Research Institute for the Advancement of Women http://criaw-icref.ca/womenhealthandaction http://criaw-icref.ca/
12. Child and Family Research Institute http://www.cfri.ca/
13. Alberta Health and Wellness: The Alberta Government Ministry of Health website, with ministry publications and a list of manuals and guidelines for health professionals: http://www.health.gov.ab.ca
14. British Columbia Centre for Health Services and Policy Research: Includes reports of various policy topics and is the repository of the former BC Office of Health Technology Assessment (BCOHTA): http://www.chspr.ubc.ca/publications
15. Effective Public Health Practice Project (EPHPP): Systematic reviews in public health produced by the Public Health Research, Education and Development (PHRED) Program, in Ontario: http://old.hamilton.ca/phcs/ephpp/ReviewsPortal.asp
16. Institute for Clinical and Evaluative Sciences (ICES): Ontario's health services and utilization research agency: http://www.ices.on.ca/
17. Manitoba Centre for Health Policy (MCHP):Manitoba's health services and utilization research agency. Source of administrative data: http://umanitoba.ca/medicine/units/mchp

18. Gov of Canada – Policy Research Initiative
    http://www.policyresearch.gc.ca/homepage.asp?pagenm=root&langcd=E

Canadian immigration/multicultural sites

1. Changing together (a centre for immigrant women) http://www.changingtogether.com/
2. Canadian heritage http://www.pch.gc.ca/eng/1266037002102/1265993639778
3. Citizenship and Immigration Canada http://www.cic.gc.ca/english/index.asp
4. Canadian Council of refuges links http://ccrweb.ca/en/links
5. Manitoba Gov's Labour and Immigration
   http://www.gov.mb.ca/msw/resources/links.html
6. Canada Updates (Making immigration simpler) http://www.canadaupdates.com/
   http://www.canadaupdates.com/content/rate-premature-birth-high-among-immigrant-women-spending-years-canada-research-16399.html
7. National Organization of Immigrant and Visible Minority Women of Canada
   http://www.noivmwc.org/
8. Metropolis http://canada.metropolis.net/

Health information

1. Canadian Institute for Health Information (CIHI) http://www.cihi.ca/
2. New Brunswick Ministry of Health Epidemiological Service. Health Publications and Services in New Brunswick http://www.gnb.ca/0051/pub/index-e.asp#epidemiology
3. Public Health Agency of Canada Reports & Publications (PHAC)
   http://www.phac-aspc.gc.ca/publications-eng.php
4. PHAC Surveillance (PHAC) http://www.phac-aspc.gc.ca/surveillance-eng.php
5. Ontario Health Coalition http://www.web.net/~ohc/
6. Canadian Academy of Health Sciences http://www.cahs-acss.ca/
7. Canadian Health Services Research foundation http://www.chsrf.ca/Splash.aspx

Immigrant Serving Organizations

1. Integration Net (by CIC) http://integration-net.ca/
2. Welcoming Communities http://welcomingcommunities.ca/
3. Ontario Community Immigrant Services Association http://ociso.org/En/
4. Alberta Association of immigrant Service Agencies http://www.aaisa.ca/
5. Mosaic BC http://www.mosaicbc.com/family-programs
6. Welcome BC
   http://www.welcomebc.ca/wbc/service_providers/programs/settlement_agencies.page
7. AMSSA http://www.amssa.org/
8. Ontario's Council on Agencies Serving Immigrants
   http://www.ocasi.org/index.php?qid=785&catid=115
9. Canadian Newcomer Magazine http://www.cnmag.ca/

Cultural organizations in Alberta

1. Catholic Social Services
   http://www.catholicsocialservices.ab.ca/CatholicSocialServices/default.aspx
2. Edmonton Mennonite Centre http://www.emcn.ab.ca/

International Health Evidence sites

1. Health Evidence Network (HEN): A WHO site that provides assessments of public health interventions for health care decision-makers: http://www.euro.who.int/en/what-we-do/data-and-evidence/health-evidence-network-hen
2. CEBM http://www.cebm.net/

Databases (Google and Google Scholar will also be searched)

1. CRD databases http://www.crd.york.ac.uk/crdweb/
2. Campbell Collaboration Library of Systematic Reviews http://www.crd.york.ac.uk/crdweb/
3. Health-Evidence.ca: Searchable online database of systematic reviews of public health and health promotion interventions geared towards decision makers: http://health-evidence.ca/articles/search
4. Evidence for Policy and Practice Information and Coordinating Centre (EPPI-Centre): provides 3 databases useful in the area of health promotion:
    - The Trials Register of Promoting Health Interventions (TRoPHI): Database of citations to health promotion-related studies and trials literature http://eppi.ioe.ac.uk/webdatabases/Intro.aspx?ID=5 .
    - Bibliomap: the EPPI-Centre database of health promotion research. All studies are coded for specific characteristics of health focus, population group and study type: http://eppi.ioe.ac.uk/webdatabases/Intro.aspx?ID=7 .
    - Database of Promoting Health Effectiveness Reviews (DoPHER)- database of health promotion reviews (mostly systematic reviews). Updated quarterly: http://eppi.ioe.ac.uk/webdatabases/Intro.aspx?ID=2 .
5. ERIC: Database of the Educational Resources Information Center includes health-related educational information: http://eric.ed.gov
6. Social Policy & Practice Database http://bathhealthnews.blogspot.com/2009/11/new-database-social-policy-practice.html

Library catalogue and book databases

1. Amicus, The Canadian National Catalogue: Includes all titles in the National Library and many other Canadian libraries. *Tip: If you have a complex search, quickly create an account and log in using the registered service so that you can do command searching*: http://www.collectionscanada.ca/amicus .
2. Theses Canada: A central access point for Canadian theses. Full text of digitized theses and dissertations are available from 1998 on: http://www.collectionscanada.gc.ca/thesescanada/
3. WorldCat: Includes over 1.4 billion items from more than 60,000 libraries worldwide. Useful to discover if the library near you has the book you are looking for: http://www.worldcat.org
4. Amazon.com: Although a commercial enterprise, Amazon is also a very large book database that is a useful tool to discover the existence of books on a particular subject. Searching is free and you might be able to find the book at your local library or through interlibrary loan: http://amazon.com

Statistics

1. Database of Online Health Statistics: Compiled by the information specialists at the Institute of Health Economics, this database provides quick and easy access to freely available web-based statistics generated by national and global agencies and research groups: http://www.ihe.ca/publications/health-db

2. Statistics Canada: Canada's national statistical agency. All electronic reports are available free of charge as of April 2006: http://www.statcan.gc.ca

Health economics sites

1. Centre for Health Economics, University of York: Research at the Centre includes economic assessments of health technologies: http://www.york.ac.uk/inst/che .
2. Centre for Health Economics and Policy Analysis (CHEPA), McMaster University: Working paper publications include many relevant to economic evaluation and health technology assessment: http://www.chepa.org
3. Institute of Health Economics (IHE): Canadian research in health economics, outcomes, policy research, and health technology assessment: http://www.ihe.ca
