# Supplementary material for: Immigrant women’s experiences of maternity-care services in Canada: a systematic review using a narrative synthesis
Source: Syst Rev. 2015 Feb 11;4:13. doi: 10.1186/2046-4053-4-13 (PMC4506414; doi:10.1186/2046-4053-4-13)
Supplement: Supplementary file 3 — Additional file 3: PRISMA 2009 Flow Diagram. (PDF 90 KB) [file 13643_2014_343_MOESM3_ESM.pdf]

PRISMA 2009 Flow Diagram

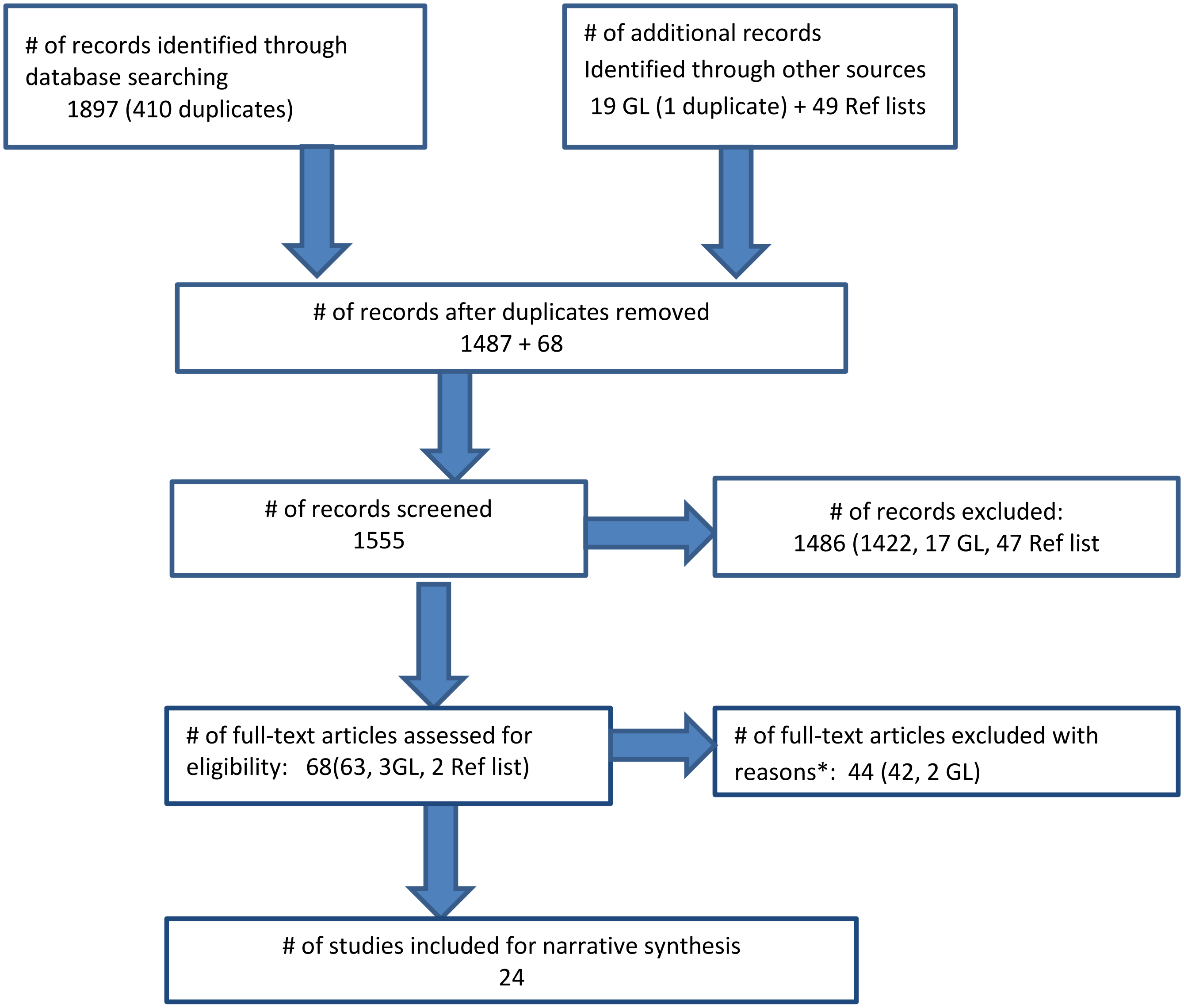

* Reasons for Exclusion for Database articles

Ref id and 1st Author	Reason for exclusion
1. 3063 Urquia	Not related to access or navigation of services
2. 3069 Janssen	Not directly related to maternity care services (violence prevention via obstetrical offices);
3. 3071 van Lieshout	Establishing validity of PPD screening tool for PPD in immigrants but nothing related to experiences in accessing care
4. 3086 Reid	Findings presented only relate to research lessons learned rather than experiences/outcomes of immigrant mothers
5. 3088 Urquia	Outcomes not related to accessing or navigating services
6. 3103 Miszkeuka	Nothing related to accessing or navigating services
7. 3121 Urquia	Nothing related to accessing or navigating services
8. 3145 Auger	Nothing related to accessing or navigating services
9. 3149 Ray	Nothing related to accessing or navigating services
10. 3159 Moore	Nothing related to accessing or navigating services
11. 3160 Urquia	Nothing related to accessing or navigating services
12. 3163 Kulig	Not specific to whether immigrants or Cana citizens
13. 3166 Small	Nothing related to accessing or navigating services
14. 3205 Quan	Nothing related to accessing or navigating services
15. 3211 Urquia	Nothing related to accessing or navigating services
16. 3321 Campbell	Knowledge and beliefs not experiences
17. 3424 Dhari	Ethnocultural beliefs and traditions but not related to actual service use or access
18. 3425 Hyman	Acculturation, social support and stress – not experiences in services
19. 3512 Hyman	Acculturation and outcomes- no experiences in services

20. 3571 Shah	Pregnancy outcomes & unrelated to service use/access
21. 3655 Vaudry	Prevalence of cCMV –nothing related to accessing and navigating
22. 3554 Larouche	Impact of imm on weight gain but not related to use or access to services
23. 3657 Fuller-Thompson	Risk factors assessed rather than preg, birth & post outcomes
24. 3774 Auger	Neighborhood & SGA – not service experiences
25. 3784 Auger	Birth outcome confounders- Nothing related to accessing or navigating services
26. 3795 Ray	Duration vs outcomes - Nothing related to accessing or navigating services
27. 3806 heaman	Immigrants only within aggregated geocoded population SED
28. 3965 Dennis	Outcomes related to imm women not related to services use or access
29. 4112 Bodo	Qualitative data not presented as empirical research and findings relate to services use were obtained from community members for which none were explicitly described as being immigrant women using health care services during maternity.
30. 4165 Crosse	Confounders of outcomes- Nothing related to accessing or navigating services
31. 4271 Urquia (thesis)	All work unrelated to access and navigation of services
32. 4421 Metchakra	Prevalence PPD & health status– not related to experiences in services
33. 4480 Groleau	? sociocultural dimensions of hyperemesis grav unrelated to services use or access
34. 4602 Zelkowitz	PPD and risk but none related to experiences with services
35. Brathwaite	About beliefs and traditions rather than experiences
36. Kurtz	Outcomes not related to accessing or navigating services

37. 4643 Edwards	Ethnocultural variables of infant care behaviours – unrelated to services use or access
38. 4735 Redwood Campbell	Data from imm women related to reproductive and other health issues (nonmaternity related) and data on prenatal care issues from sponsor groups not women
39. 4746 Davey	One comment in paper about service prediction of PPD (prenatal support not affecting rates of PPD) but not specific to immigrant women and sample only 25% immigrant.
40. 4796 Zelkowitz	PPD and variables of its continuation but none related top services use/access
41. 4884 Zelkowitz	Risk factors for PPD but none related to services use/access
42. 4899 Jimenez	Prevalence of hyperemesis but unrelated to services experiences

No for Grey Literature:

1. 7 –PPP Precarious status	Not directly related to maternity care services
2. 132 thesis- Quality of Prenatal care	Not directly related to maternity care services
