# Supplementary material for: Immigrant women’s experiences of maternity-care services in Canada: a systematic review using a narrative synthesis
Source: Syst Rev. 2015 Feb 11;4:13. doi: 10.1186/2046-4053-4-13 (PMC4506414; doi:10.1186/2046-4053-4-13)
Supplement: Supplementary file 4 — Additional file 4: Search Strategy overview and table for MEDLINE: immigrant women’s maternity-care experiences in Canada. (PDF 81 KB) [file 13643_2014_343_MOESM4_ESM.pdf]

Additional file 4: Search Strategy overview and table for MEDLINE: Immigrant women's maternity care experiences in Canada

The following databases will be searched: Ovid Medline In process and 1948-, Ovid Embase, Ovid PsycINFO, EBSCOhost CINAHL, Thompson Reuters Web of Science, Scopus 1960-, and CSA Sociological Abstracts. Databases were chosen based upon their coverage of the health sciences (Medline, Embase, PsycINFO, CINAHL) and their inter-disciplinary coverage (Web of Science, Scopus, Sociological Abstracts) since this topic may be of interest to health and social science researchers. The search strategy was designed and implemented by a health sciences librarian (TC). The search strategy had six main concepts: maternal health services, accessibility to health services for pregnant women, cultural appropriateness of health services, pregnancy and birth outcomes, immigrants, and Canada. A search strategy using appropriate commands, subject headings, and key words was developed and adapted for each of the databases. No language restrictions were used in the search so that both English and French language items would be retrieved. Only literature published from 1995 onwards was retrieved.

Strategy for MEDLINE

**Platform**	**Ovid**
**Database**	**MEDLINE(R) In-Process & Other Non-Indexed Citations, Ovid MEDLINE(R) Daily and Ovid MEDLINE(R) 1948 to Present**
**Search run**	November 28, 2011
**Search strategy**	1. maternal health services/ or postnatal care/ or preconception care/ or prenatal care/<br>2. ((birth* or matern* or mother* or prenatal or pre-natal or postnatal or post-natal or preconception or pre conception or antenatal or ante-natal or postpartum or puerperium or perinatal) adj3 (care or service*)).tw.<br>3. 1 or 2<br>4. Health Services Accessibility/<br>5. Healthcare Disparities/<br>6. Health Literacy/<br>7. (access* adj3 (health service* or health care or healthcare)).tw.<br>8. (availab* adj3 (health service* or health care or healthcare)).tw.<br>9. (barrier* adj3 (health service* or health care or healthcare)).tw.<br>10. (facilitat* adj3 (health service* or health care or healthcare)).tw.<br>11. (usage adj3 (health service* or health care or healthcare)).tw.<br>12. (utili?ation adj3 (health service* or health care or healthcare)).tw.<br>13. ("use" adj3 (health service* or health care or healthcare)).tw.

	14. (disparit* adj3 (health service* or health care or healthcare)).tw.<br>15. (patient adj3 navigat*).tw.<br>16. or/4-15<br>17. exp Pregnancy/<br>18. (pregnan* or childbearing or child bearing or pre-natal or postnatal or post-natal or preconception or pre conception or antenatal or ante-natal or postpartum or puerperium or perinatal).tw.<br>19. 17 or 18<br>20. 16 and 19<br>21. Cultural Competency/ or Prejudice/ or "Cultural Diversity"/ or "Transcultural Nursing"/ or cross-cultural comparison/ or cultural characteristics/<br>22. ((cultur* adj appropriate*) or (cultural competenc* or prejudice* or racist* or racism or bigot*)).tw.<br>23. 21 or 22<br>24. 3 or 19<br>25. 23 and 24<br>26. Infant Mortality/ or Maternal Mortality/ or Perinatal Mortality/ or Pregnancy outcome/ or Fetal Death/<br>27. infant, low birth weight/ or infant, small for gestational age/ or infant, very low birth weight/ or infant, extremely low birth weight/ or infant, premature/<br>28. abortion, spontaneous/ or diabetes, gestational/ or fetal macrosomia/ or fetal membranes, premature rupture/ or chorioamnionitis/ or obstetric labor, premature/ or premature birth/ or Fetal Alcohol Syndrome/<br>29. Depression, Postpartum/ or exp Congenital Abnormalities/ or exp Fetal Diseases/ or delivery, obstetric/ or cesarean section/ or cesarean section, repeat/ or exp Obstetric Labor Complications/<br>30. (exp Hemoglobinopathies/ or exp Anemia/ or Smoking/) and pregnancy/<br>31. (mortality adj1 (infant* or maternal or perinatal or prenatal)).tw.<br>32. (pre term birth* or preterm birth* or PROM or gestational diabet* or premature birth* or stillborn or stillbirth*).tw.<br>33. (healthy adj3 (pregnancy or labo?r or delivery or birth or fetal outcome*)).tw.<br>34. (normal adj3 (pregnancy or labo?r or delivery or birth or fetal outcome*)).tw.<br>35. (miscarr* or postpartum depression or vaginal birth or caesarean or c-section*).tw.<br>36. ((h?emoglobinapath* or an?emia or smok*) and pregnan*).tw.<br>37. or/26-35<br>38. "emigrants and immigrants"/ or refugees/ or "transients and migrants"/ or "Emigration and Immigration"/<br>39. (immigrat* or immigrant* or refugee* or newcomer* or new-comer* or alien or aliens or adoptive citizen* or foreigner* or incomer* or naturalized citizen* or foreign born or country of birth or migrant farm worker* or temporary foreign worker*).tw.<br>40. african continental ancestry group/ or african americans/ or asian continental ancestry group/ or asian americans/ or arabs/ or exp hispanic americans/<br>41. (south asian* or indo-canadian or chinese or filipino or filipina or phillipines or ital* or poland or polish or vietnam or vietnamese or portugal or portuguese

	or pakistan* or korea* or sri lanka* or mexican or mexico or russia* or iran* or nigeria* or south africa* or brazil* or saudi arabia*).tw.<br>42. or/38-41<br>43. 3 and 42<br>44. 20 and 42<br>45. 37 and 42<br>46. 25 or 43 or 44 or 45<br>47. exp canada/<br>48. canada.cp.<br>49. (canada or canadian$ or alberta or british columbia or columbie britannique).af.<br>50. (saskatchewan or manitoba or ontario or quebec or new brunswick or nouveau brunswick).af.<br>51. (nova scotia or nouvelle ecosse or prince edward island or ile du prince edward or newfoundland or terre neuve or labrador or nun?v?t or nun?v?t or nwt or territoires du nord ouest or northwest territories or yukon).af.<br>52. (canada or canadian$ or alberta or british columbia or columbie britannique).in,jw,nw,jx.<br>53. (saskatchewan or manitoba or ontario or quebec or new brunswick or nouveau brunswick).in,jw,nw,jx.<br>54. (nova scotia or nouvelle ecosse or prince edward island or ile du prince edward or newfoundland or labrador or nun?v?t or nwt or northwest territories or territoires du nord ouest or yukon).in,jw,nw,jx.<br>55. or/47-54<br>56. 46 and 55<br>57. limit 56 to yr="1995 -Current"
